# Blocking the recruitment of naive CD4^+^ T cells reverses immunosuppression in breast cancer

**DOI:** 10.1038/cr.2017.34

**Published:** 2017-03-14

**Authors:** Shicheng Su, Jianyou Liao, Jiang Liu, Di Huang, Chonghua He, Fei Chen, LinBing Yang, Wei Wu, Jianing Chen, Ling Lin, Yunjie Zeng, Nengtai Ouyang, Xiuying Cui, Herui Yao, Fengxi Su, Jian-dong Huang, Judy Lieberman, Qiang Liu, Erwei Song

**Affiliations:** 1 Guangdong Provincial Key Laboratory of Malignant Tumor Epigenetics and Gene Regulation, Medical Research Center, Sun Yat-Sen Memorial Hospital, Sun Yat-Sen University, Guangzhou, Guangdong 510120, China; 2 Breast Tumor Center, Sun Yat-Sen Memorial Hospital, Sun Yat-Sen University, Guangzhou, Guangdong 510120, China; 3 Department of Internal Medicine, The First Affiliated Hospital, Shantou University Medical College, Shantou, Guangdong 515041, China; 4 Department of Pathology, Sun Yat-Sen Memorial Hospital, Sun Yat-Sen University, Guangzhou, Guangdong 510120, China; 5 Department of Biochemistry, the University of Hong Kong, Hong Kong, SAR, China; 6 Department of Pediatrics, Program in Cellular and Molecular Medicine, Boston Children's Hospital, Harvard Medical School, Boston, MA, USA

**Keywords:** naive CD4^+^ T cells, breast cancer, Tregs, CCL18, tumor immunosuppression

## Abstract

The origin of tumor-infiltrating Tregs, critical mediators of tumor immunosuppression, is unclear. Here, we show that tumor-infiltrating naive CD4^+^ T cells and Tregs in human breast cancer have overlapping TCR repertoires, while hardly overlap with circulating Tregs, suggesting that intratumoral Tregs mainly develop from naive T cells *in situ* rather than from recruited Tregs. Furthermore, the abundance of naive CD4^+^ T cells and Tregs is closely correlated, both indicating poor prognosis for breast cancer patients. Naive CD4^+^ T cells adhere to tumor slices in proportion to the abundance of CCL18-producing macrophages. Moreover, adoptively transferred human naive CD4^+^ T cells infiltrate human breast cancer orthotopic xenografts in a CCL18-dependent manner. In human breast cancer xenografts in humanized mice, blocking the recruitment of naive CD4^+^ T cells into tumor by knocking down the expression of PITPNM3, a CCL18 receptor, significantly reduces intratumoral Tregs and inhibits tumor progression. These findings suggest that breast tumor-infiltrating Tregs arise from chemotaxis of circulating naive CD4^+^ T cells that differentiate into Tregs *in situ*. Inhibiting naive CD4^+^ T cell recruitment into tumors by interfering with PITPNM3 recognition of CCL18 may be an attractive strategy for anticancer immunotherapy.

## Introduction

Regulatory T cells (Tregs), a subset of immunosuppressive Foxp3^+^CD25^+^CD4^+^ T cells that play an important role in maintaining self-tolerance, are enriched in many cancers. Tregs suppress the anti-tumor function of effector T cells and natural killer cells by secreting soluble immunosuppressive factors, such as TGF-β, and expressing inhibitory receptors such as CTLA4^1,2^. A high proportion of Tregs in tumor-infiltrating (TI) T cells in cancer specimens from many types of human cancer is associated with poor prognosis^3,4^. Elimination of Tregs with an anti-CD25-coupled toxin has shown promise in early-phase clinical trials^5,6^. Animal studies also suggest that Tregs play a pivotal role in tumor immune escape^2^. The clinical benefit of checkpoint blockade with anti-CTLA4 has also been partially attributed to depletion of TI Tregs^7^. Together, these results suggest that Tregs are promising targets for anti-tumor immunotherapy. However, systemic suppression of Tregs can induce severe autoimmune complications^8^. Therefore, strategies to selectively deplete TI Tregs without interfering with systemic immune homeostasis would be ideal for tumor immunotherapy. However, developing such strategies requires an understanding of the source of TI Tregs and what distinguishes TI Tregs from circulating Tregs and Tregs at normal tissue sites. Surprisingly, little is known about how TI Tregs arise^1,3,9^.

The goal of this study is to understand how human breast TI Tregs are generated. The prevailing idea is that TI Tregs are recruited from preexisting circulating Tregs by chemokines or chemokine ligands expressed by tumor cells, stroma or tumor-associated macrophages (TAMs). Several chemokine-chemokine receptor pairs have been proposed to be responsible for recruiting Tregs to tumors including chemokine receptors CCR4, CCR10, CCR8 or CXCR3 expressed on infiltrating Tregs, responding to CCL22, CCL28, CCL1 or CXCL9-11 in the tumor microenvironment, respectively^10,11,12^. An alternative possibility is that naive or conventional T cells might be recruited to the tumor and differentiate to Tregs *in situ* within the immunosuppressive tumor environment^1,9,13^. Here we show that the TAM-produced chemokine CCL18 recruits circulating naive CD4^+^ T cells to breast tumors by binding their PITPNM3 receptor. Furthermore, TCR repertoire analysis suggests that Tregs in human breast cancer are mainly derived from naive CD4^+^ T cells. In human breast cancer xenograft models, blocking CCL18-mediated recruitment of naive T cells into tumors reduces TI Tregs and inhibits tumor progression.

## Results

### The TCR clonotypes of breast TI Tregs most closely resemble those of naive CD4^+^ T cells in the tumor and peripheral blood

**Figure 1:**
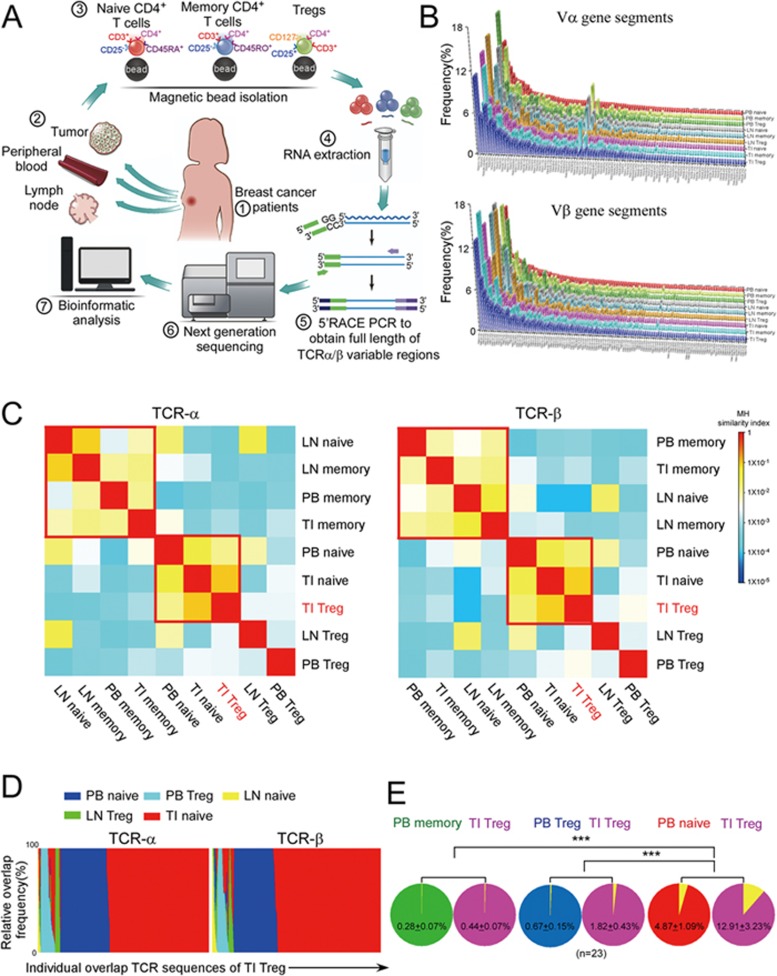
The TCR repertoire of breast cancer tumor-infiltrating Tregs is most similar to that of naive CD4^+^ T cells. **(A-D)** Full-length TCR-β/α variable regions of Tregs, naive CD4^+^ T cells and memory CD4^+^ T cells from peripheral blood (PB), lymph nodes (LN) and primary tumors (tumor-infiltrating, TI) of five breast cancer patients were amplified and sequenced. Pooled data from all five patients were compared. **(A)** Experimental schematic. **(B)** Frequencies of *Vα/β* gene usage in the groups of isolated T cells (*V* genes were ordered based on decreasing frequency in PB naive CD4^+^ T cells). **(C)** Similarity of pooled TCR repertoires, calculated using the Morisita-Horn similarity index^16^, was used to cluster the groups of T cells analyzed. A value between 0 (no similarity) and 1 (identical) was calculated and colored according to the shown scale. **(D)** Individual overlap sequences of TI Treg identified in other groups of T cells. Individual TCR sequences of TI Tregs were arrayed on the *x* axis and the relative frequency at which this particular sequence was found in other subsets was plotted on the *y* axis. **(E)** Proportion of unique TRBV12-4/TRBJ1-2 sequences shared between TI Tregs and the 3 PB T cell subtypes (Treg, naive and memory CD4 T cells) in 23 breast cancer patient samples (shown are proportion of overlapping sequences (mean ± SEM), ^***^P < 0.001 by Student's *t*-test).

To begin to evaluate whether Tregs in breast tumors are mainly recruited from peripheral blood (PB) or converted *in situ* from other CD4^+^ lymphocyte subtypes, we isolated Tregs (CD4^+^CD25^+^CD127^−/low^), naive CD4^+^ T cells (CD4^+^CD45RA^+^CD25^−^) and memory CD4^+^ T cells (CD4^+^CD45RO^+^CD25^−^) from breast cancers (tumor infiltrating, TI), PB and ipsilateral draining lymph nodes (LN) of 5 breast cancer patients and used next-generation sequencing to compare their TCR-β/α repertoires (Figure 1A). The purity of the isolated cell populations was > 90% (Supplementary information, Figure S1). 5′ rapid amplification of cDNA ends (RACE) PCR was used to amplify the full-length variable regions of all *TCR-β/α* genes in each sample. The mapped reads showed grossly similar *TCR-β/α* gene usage in TI, PB and LN T cells (Figure 1B and Supplementary information, Figure S2), consistent with a recent report on TI T cell repertoire based on data in the Cancer Genome Atlas^14^.

To look for more subtle differences, we pooled the TCR sequencing data for each of the nine sources of CD4^+^ T cell subsets (Treg, naive and memory CD4^+^ T cells from tumor, LN and blood) from all five patients^15^ and used unsupervised clustering to compare their TCR-α and TCR-β usage^16^. For both subunits, *TCR* gene usage in TI Tregs was most similar to that of TI and PB naive CD4^+^ T cells, whereas gene usage of TI memory CD4^+^ T cells clustered with PB and LN memory CD4^+^ T cells, and LN naive CD4^+^ T cells (Figure 1C). Next we studied what proportion of the individual TCR sequences of TI Tregs was identical to those from the other groups^17^. The TCR sequences of both TCR-β and -α from TI Tregs overlapped most with the sequences of TI naive CD4^+^ T cells and then with PB naive CD4^+^T cells (Figure 1D).

The TCR sequences of TI Tregs and the same subsets of PB CD4^+^ T cells were compared in a larger group of 23 breast cancer patients by studying the TCR sequences of a single *V_β_J_β_* gene rearrangement (TRBV12-4/TRBJ1-2). Within this set of gene rearrangements, 12.9% ± 3.2% of the TI Treg sequences overlapped with 4.9% ± 1.1% of the CD4^+^ naive T cell sequences, whereas a significantly much smaller proportion of clonotypic sequences (< 2%) was identical between TI Tregs and PB Tregs or CD4^+^ memory T cells (Figure 1E). Thus the TCR clonotypes of TI Tregs are most similar to those of TI and PB naive CD4^+^ T cells, suggesting that TI Tregs might develop from naive CD4^+^ T cells recruited from the blood.

### The abundance of naive CD4^+^ T cells in breast cancer samples is associated with increased TI Tregs and poor prognosis

**Figure 2:**
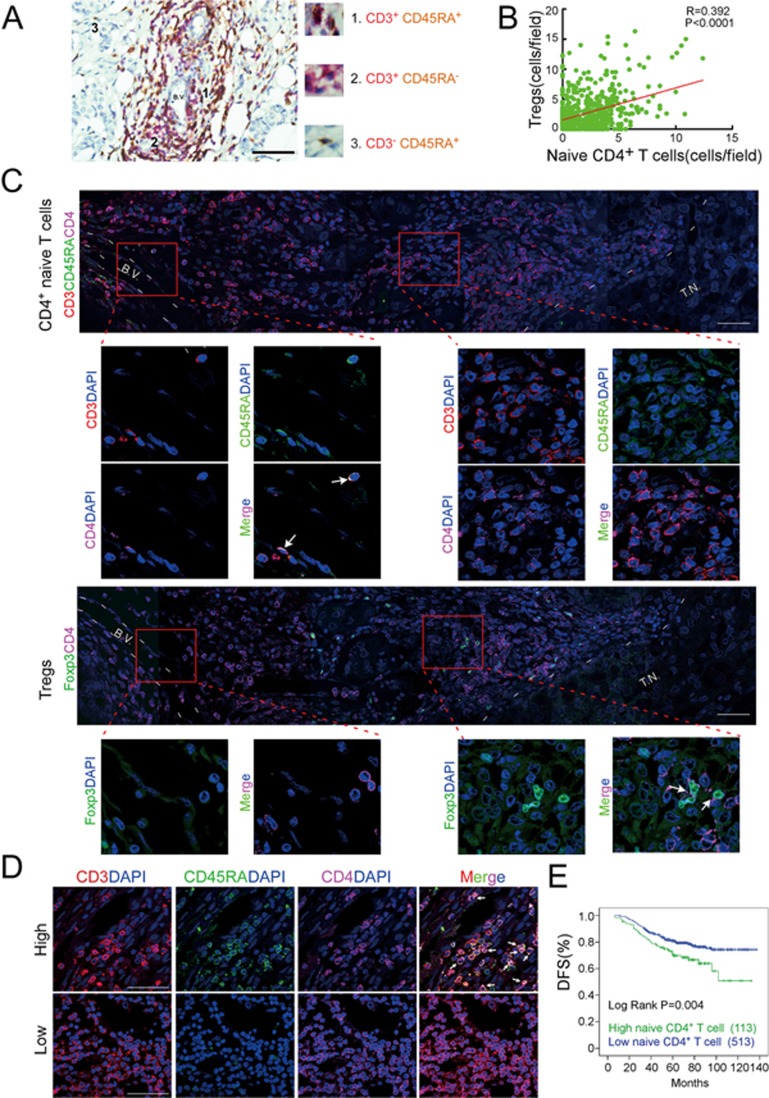
Naive CD4^+^ T cell abundance within breast tumors is associated with increased numbers of Tregs and poor patient prognosis. **(A)** IHC staining of CD3 (red) and CD45RA (brown) in a representative breast cancer sample. Naive T cells were defined as CD3^+^CD45^+^ cells. Scale bar, 50 μm. **(B)** Correlation of TI naive CD4^+^ T cell numbers and TI Treg numbers in breast cancer samples (*n* = 626, Pearson correlation coefficient *R* and *P*-value are shown). **(C)** Representative immunofluorescent staining for naive T cells (CD3 (red), CD45RA (green) and CD4 (purple); upper panels) or Tregs (Foxp3 (green) and CD4 (purple); lower panels) in serial sections from a human breast cancer sample. Arrows indicate CD3^+^CD4^+^CD45RA^+^ naive CD4^+^ T cells (upper) and CD4^+^Foxp3^+^ Tregs (lower). Scale bar, 50 μm. The localization of naive CD4 T cells and Tregs relative to the perivascular space or tumor parenchyma for 626 tumor samples is provided in Supplementary information, Figure S3C. **(D)** Representative immunofluorescent staining of CD3 (red), CD45RA (green), CD4 (purple) and DAPI (blue) in breast cancer samples with high (upper panel) or low (lower panel) number of naive CD4^+^ T cells, which are indicated by arrows. Scale bar, 50 μm. **(E)** Kaplan-Meier survival curve of breast cancer patients with low and high numbers of TI naive CD4^+^ T cells. BV, blood vessel; TN, tumor nest.

We next evaluated by immunohistochemistry (IHC) whether we could detect naive T cells within clinical breast cancer tissues. CD3^+^CD45RA^+^ naive T cells were detected in 74% (462 of 626 cases) of clinical breast cancer tissues, predominantly in the perivascular space (Figure 2A). The number of naive CD4^+^ T cells positively correlated with the numbers of Tregs in tumors (*P* < 0.0001; Figure 2B). In contrast, the number of memory CD4^+^ T cells (CD4^+^CD45RO^+^Foxp3^−^) in tumors negatively correlated with the number of Tregs and naive CD4^+^ T cells in the slides (Supplementary information, Figure S3A). We also used immunofluorescence microscopy to analyze serial breast tumor sections stained for CD3 and CD4, and either CD45RA (naive CD4^+^ T cells) or Foxp3 (Tregs). Naive CD4^+^ T cells were more prominent in the perivascular space, whereas the majority of Tregs were far from blood vessels and localized close to the tumor parenchyma (Figure 2C, Supplementary information, Figure S3B and S3C). These data are consistent with the possibility that naive CD4^+^ T cells entering from the blood differentiate into Tregs after they migrate toward the tumor.

We next evaluated whether a high number of TI naive CD4^+^ T cells has prognostic significance. We used X-Tile software to separate the 626 patient samples into one group (113 patients) with a high number of TI naive CD4^+^ T cells (> 3.8 cells per field) and another group (513) with a lower number. The patients with more TI CD4^+^ naive T cells had significantly reduced disease-free survival (*P* < 0.004; Figure 2D and 2E). By contrast, a parallel analysis showed that naive CD8^+^ T cell abundance did not correlate with disease-free survival (Supplementary information, Figure S3D and S3E). A significantly higher TI naive CD4^+^ T cell count was associated with other indicators of poor prognosis — triple-negative breast cancer subtype, lymphovascular invasion, LN metastasis and distant metastases (Supplementary information, Table S1). Multivariate analysis demonstrated that the abundance of TI naive CD4^+^ T cells was an independent prognostic factor (Supplementary information, Table S2). In addition, a higher number of TI memory CD4^+^ T cells was significantly associated with good prognosis (Supplementary information, Figure S3F), whereas Treg accumulation was associated with poor prognosis (Supplementary information, Figure S3G).

To confirm the clinical significance of TI naive CD4^+^ T cells, we stained tumor sections for another naive T cell marker, CD62L^18^ (Supplementary information, Figure S4A). CD3^+^CD4^+^CD62L^+^ cell counts significantly correlated with CD3^+^CD4^+^CD45RA^+^ cell counts (Supplementary information, Figure S4B) and were also significantly associated with poor prognosis (Supplementary information, Figure S4C). To confirm the association of naive CD4^+^ T cells with poor prognosis in independent large cohorts, we analyzed the correlation of CD4^+^ naive T cell-associated gene expression with clinical prognosis in the Curtis and Hatzis data sets, two of the largest breast cancer case lists in the online database Oncomine. Because data on CD45RA expression and some clinical parameters are not available in Oncomine, we analyzed the correlation between the annotated clinical variables with expression of CD4 and CD62L. Patients in each data set were divided into two groups with or without high expression of both CD4 and CD62L using X-Tile. High expression of both genes was significantly associated with increased LN metastasis in both data sets, an increase in triple-negative subtypes in the Curtis data set and distant metastasis, and poor long-term metastasis-free survival in the Hatzis data set (Supplementary information, Table S3 and Figure S4D). Thus a high number of TI naive CD4^+^ T cells are associated with poor prognosis.

### Naive CD4^+^ T cells convert to functional Tregs in a breast cancer microenvironment *in vitro*

**Figure 3:**
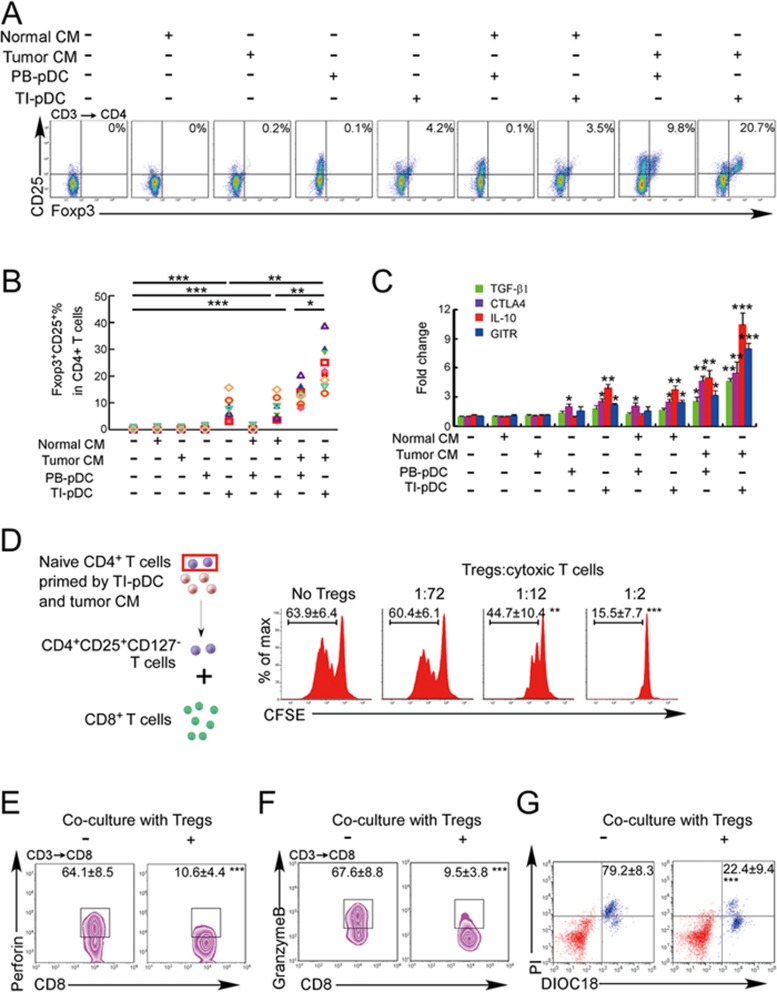
Naive CD4^+^ T cells are converted to functional Tregs by tumor-infiltrating DCs and tumor conditioned medium (CM). **(A-C)** Naive CD4^+^ T cells from peripheral blood of patients with invasive breast carcinoma were co-cultured with or without autologous pDCs isolated from tumor (TI) or peripheral blood (PB) for 9 days in the presence or absence of 30% CM from autologous tumor slices or adjacent normal tissue slices. **(A**, **B)** Non-adherent cells from co-cultures were stained for CD3, CD4, CD25 and intracellular Foxp3, and analyzed by flow cytometry. Representative plots of gated CD3^+^CD4^+^ cells **(A)** and quantification of percentage of Foxp3^+^CD25^+^ cells among CD3^+^CD4^+^ cells **(B)** are shown (mean ± SEM, *n* = 19; ^*^P < 0.05, ^**^P < 0.01,^***^P < 0.001 by Student's *t*-test). **(C)** Expression of Treg-associated genes, assessed by qRT-PCR normalized to *GAPDH*, in sorted CD4^+^ T cells, relative to expression in cultures without DCs or CM (mean ± SEM, *n* = 19; ^*^P < 0.05, ^**^P < 0.01,^***^P < 0.001 compared with naive CD4^+^ T cells cultured alone by Student's *t*-test). **(D-G)** Effect of naive CD4^+^ T cell-derived Tregs, obtained by co-culture with TI pDCs and tumor CM as above, on function of autologous tumor-specific CD8^+^ T cells. Tumor-specific CD8^+^ T cells were generated for each subject by stimulating autologous PB CD8^+^ T cells with autologous tumor lysate-pulsed autologous DCs. Tregs were recovered from co-cultures by magnetic sorting. **(D)** CFSE-labeled CD8^+^ T cells were incubated with tumor lysate-pulsed DCs in the presence of induced Tregs at the indicated ratios and proliferation was assessed by flow cytometry. Numbers denote the percentage of cells undergoing at least one cellular division (mean ± SEM, *n* = 12, ^**^P < 0.01, ^***^P < 0.001 compared with CD8^+^ T cells cultured without Tregs). **(E-G)** Tumor-specific CD8^+^ T cells were incubated with autologous primary breast cancer cells for 18 h in the presence or absence of Tregs (CD8:Treg 2:1) and stained for CD3, CD8, intracellular perforin **(E)** or granzyme B **(F)** and gated CD3^+^CD8^+^ cells were analyzed by flow cytometry. Numbers indicate the percentage of gated cells stained for perforin or granzyme B (mean ± SEM, *n* = 7; ^***^P < 0.001 compared with CD8^+^ T cells cultured without Tregs). **(G)** Tumor-specific CD8^+^ T cells were incubated with autologous dioctadecyloxacarbocyanine (DIOC18)-labeled primary breast cancer cells for 18 h in the presence or absence of Tregs (CD8:Treg 2:1) and the death of tumor cells was assessed by propidium iodide (PI) uptake by flow cytometry. The numbers shown indicate the percentage of PI^+^ tumor cells (mean ± SEM, *n* = 4; ^***^P < 0.001 compared with CD8^+^ T cells cultured without Tregs).

Our data so far suggest that naive CD4^+^ T cells may differentiate into Tregs within breast tumors. Dendritic cells (DCs) have been shown to induce naive T cell conversion into Tregs in the presence of cancer cell-secreted immunomodulators^19,20^. To determine whether naive CD4^+^ T cells can develop into Tregs in the tumor microenvironment^13^, we co-cultured naive CD4^+^ T cells, isolated from the PB of breast cancer patients, with autologous plasmacytoid DCs (pDCs), isolated from the blood or breast cancer tissues in normal culture medium or medium that was supplemented with 30% conditioned medium (CM) from autologous cancer tissue slices (Figure 3A and 3B). TI pDCs, but not PB pDCs, converted 7.5% ± 1.7% of naive CD4^+^ T cells to CD25^+^Foxp3^+^ Tregs in unsupplemented medium. Tumor CM on its own without DCs did not induce Treg differentiation of naive CD4^+^ T cells, but PB pDCs caused Treg differentiation when tumor CM was added. However, the greatest effect, 23.2% ± 3.4% Tregs, was seen when both TI pDCs and tumor CM were added. The co-cultures that increased the numbers of Tregs, defined by CD25 and Foxp3 expression, also increased the mRNA expression of Treg-associated genes, *TGFB1*, *CTLA4*, *IL10* and *GITR* by quantitative reverse transcription (qRT)-PCR (Figure 3C). Increases in expression of these genes were in proportion to the generation of CD25^+^Foxp3^+^ cells in the cultures, being greatest for naive cells cultured in the presence of both TI pDCs and CM. We observed similar results when myeloid DCs were substituted for pDCs (Supplementary information, Figure S5).

To determine whether the Tregs converted from naive CD4^+^ T cells are functional immunosuppressor cells, we isolated CD25^+^CD127^−^ Tregs from the co-cultures and evaluated their ability to suppress autologous CD8^+^ cytotoxic T lymphocytes (CTLs) (Figure 3D-3G). Adding the induced Tregs strongly inhibited proliferation of CTLs in response to tumor lysate-pulsed autologous DCs in proportion to the numbers of Tregs added (Figure 3D), and markedly reduced their perforin (Figure 3E) and granzyme B (Figure 3F) levels. CTL killing of autologous primary breast cancer cells, assessed by propidium iodide staining and loss of mitochondrial transmembrane potential, was significantly reduced after co-culture with the induced Tregs (Figure 3G). These *in vitro*-generated Tregs also similarly functionally suppressed tumor-associated antigen (Muc1)-specific CTLs, generated from CTLs by exposure to Muc1 peptide-incubated autologous DCs (Supplementary information, Figure S6). Thus naive CD4^+^ T cells can be converted to functional Tregs *in vitro* by exposure to TI DCs and tumor CM.

### Breast cancers express more CCL18 than adjacent normal breast tissues

**Figure 4:**
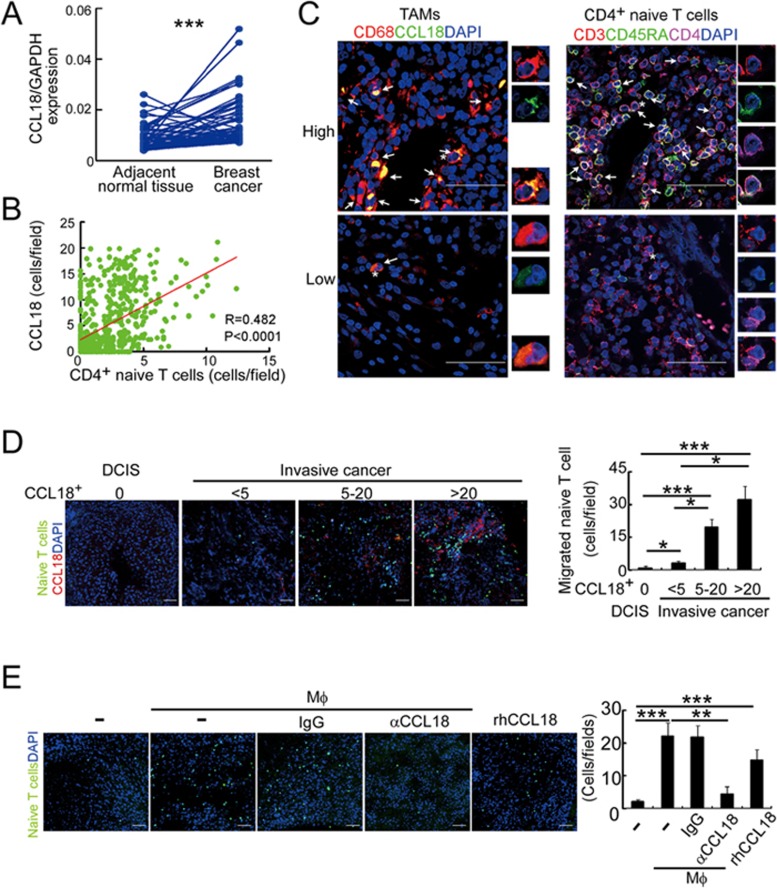
Naive CD4^+^ T cells are recruited to breast tumors by TAM-secreted CCL18. **(A)** CCL18 expression in breast cancer tissues and paired adjacent normal breast tissues detected by qRT-PCR relative to *GAPDH*(*n* = 52; ^***^P < 0.001 by Student's *t*-test). **(B)** Correlation of numbers of CCL18^+^ TAM (CD68^+^CCL18^+^) and naive CD4^+^ T (CD3^+^CD4^+^CD45RA^+^) cells in breast cancer samples (*n* = 626, Pearson's correlation coefficient *R* and *P*-value are shown). **(C)** Representative immunofluorescent staining for CD68 and CCL18 and CD3, CD45RA and CD4 from CCL18-high (upper panel) and CCL18-low (lower panel) breast cancer samples. CCL18^+^ TAMs and naive CD4^+^ T cells are indicated by arrows. Asterisk indicates the location of higher magnification images at right. Scale bar, 50 μm. **(D)** Naive PB CD4^+^ T cells labeled with CFSE (green) were overlaid on autologous breast tumor slices that were then fixed and stained for CCL18 (red). Representative images (left) and quantification of number of adherent CFSE^+^ T cells (mean ± SEM; right) are shown (ductal carcinoma *in situ* (DCIS), *n* = 4; invasive cancer with CCL18^+^ cell count < 5, *n* = 9; 5-20, *n* = 6; > 20, *n* = 4; ^*^P < 0.05; ^***^P < 0.001 by Student's *t*-test). Scale bar, 50 μm. **(E)** CFSE-labeled PB naive CD4^+^ T cells from healthy donors were intravenously injected via tail vein, with or without control IgG or CCL18-neutralizing antibody, into NOD/scid mice bearing subcutaneous MDA-MB-231 breast cancers that were implanted 14 days earlier either alone or with autologous human macrophages. In some mice, the xenografts were injected with rhCCL18. The number of CFSE^+^ T cells that migrated into the xenografts was measured by immunofluorescence microscopy 48 h after T cells were injected. Shown are representative images (left) and the number of CFSE^+^ cells/high power field for each condition (mean ± SEM, *n* = 8 mice per group. ^**^P < 0.01; ^***^P < 0.001 by Student's *t*-test). Scale bar, 50 μm.

Our data suggest that naive CD4^+^ T cells are recruited from the blood to breast tumors, where they can differentiate into immunosuppressive Tregs. Next we wanted to know the mechanism responsible for naive CD4^+^ T cell recruitment. As T cell migration is orchestrated by responses to chemokine gradients, we first compared the relative mRNA expression of a panel of chemokines to which naive T cells respond — CCL3, 4, 18, 19 and 21 — in 52 breast cancers vs paired adjacent normal breast tissues. Of these chemokines, only CCL18 mRNA was significantly higher in breast cancer than the paired adjacent normal tissue (Figure 4A and Supplementary information, Figure S7A). In five independent breast cancer online data sets, CCL18 mRNA was increased in the tumor relative to normal tissue and significantly increased in two data sets (Supplementary information, Table S4). TAMs abundantly express CCL18^21^, a chemokine toward which naive T cells preferentially migrate^22^. In our samples, the mRNAs of CCL22 and CCL28, two chemokines to which Tregs can respond^4,10^, did not increase and CCL28 even decreased in tumor (Supplementary information, Figure S7A). Significant changes in CCL22 and CCL28 in the online breast cancer data sets also showed decreased tumor expression (Supplementary information, Table S4), suggesting that these chemokines are unlikely to play an important role in Treg recruitment to breast cancer. Based on expression data, CCL18 is an attractive candidate chemokine for recruiting naive CD4^+^ T cells to breast tumors.

### CCL18, expressed on TAMs, preferentially recruits naive CD4^+^ T cells to breast tumors *in vitro* and *in vivo*

To explore a potential role for CCL18, we used immunofluorescence microscopy to examine whether CCL18 expression on TAMs correlates with naive CD4^+^ T cells in tumors. 626 breast cancer samples were stained for CD3, CD4 and CD45RA to identify naive CD4^+^T cells and for CD68 and CCL18 to identify CCL18^+^ TAMs (Figure 4B and 4C). The numbers of naive CD4^+^ T cells and CCL18^+^ TAMs were highly correlated (*P* < 0.0001). To determine whether CCL18^+^ TAMs could recruit naive T cells to tumors, we used a tumor slice assay in which freshly resected breast cancer slices were overlaid with autologous naive CD4^+^ T cells labeled with carboxyfluorescein succinimidyl ester (CFSE), and the number of stained CD4^+^ T cells bound was counted and compared to the numbers of CCL18^+^ TAMs in the tumor^23^. As controls, we treated slides from patients with DCIS. Naive CD4^+^ T cells adhered to the tumor slices in proportion to the number of CCL18^+^ TAMs (Figure 4D). Significantly more naive CD4^+^ T cells adhered to all the cancer samples, even those with the least numbers of CCL18^+^ TAMs, compared to the DCIS samples.

Next we wanted to determine whether CCL18^+^ TAMs recruit naive CD4^+^ T cells from the blood *in vivo*. MDA-MB-231, a triple-negative breast cancer cell line, induces macrophages to produce CCL18^24^. We therefore examined *in vivo* recruitment^25^ of intravenously injected CFSE-labeled human normal donor naive CD4^+^ T cells into palpable mammary fat pad breast tumors in NOD/scid mice, formed by MDA-MB-231 breast cancer cells, co-injected with or without human macrophages (Figure 4E). Xenografts formed with macrophage co-injection showed increased naive CD4^+^ T cell infiltration. In some experiments the CD4^+^ T cells were co-injected with anti-CCL18 or isotype-matched control antibody. Blocking CCL18 reduced CD4^+^ T cell localization to the xenograft, almost to the level of xenografts that were formed without macrophage co-injection. Moreover, intratumoral injection of recombinant CCL18 in orthotopic xenografts formed without macrophages effectively substituted for the macrophages to promote naive CD4^+^ T cell recruitment. Thus CCL18 expression by TAMs recruits naive CD4^+^ T cells to orthotopic breast cancer xenografts. It should be noted that there were no Foxp3^+^ Tregs in any of the xenografts (Supplementary information, Figure S7B) and no difference in tumor size among different groups (Supplementary information, Figure S7C). This finding is not unexpected, as human DCs, which prime the generation of Tregs, are absent from this model.

To investigate whether CCL18 also recruits memory CD4^+^ T cells or Tregs, we repeated these experiments substituting intravenously injected memory CD4^+^ T cells or Tregs for the naive CD4^+^ T cells (Supplementary information, Figure S7D and S7E). Memory CD4^+^ T cell recruitment was not affected by macrophage co-injection or anti-CCL18, whereas Treg recruitment was enhanced by macrophage co-injection, but independently of CCL18. Moreover, recombinant CCL18 did not increase Treg chemotaxis in a transwell assay, whereas the known Treg migration mediator CCL22 did (Supplementary information, Figure S7F). Thus CCL18 expressed by TAMs selectively recruits circulating naive CD4^+^ T cells, but not memory or Treg CD4^+^ T cells, into breast cancer. However, incubation of naive healthy donor CD4^+^ T cells with CCL18 did not induce CD25 or Foxp3 expression or confer on them the ability to suppress the proliferation of autologous CD8^+^ T cells that were stimulated with MDA-MB-231 cell lysate-pulsed myeloid DCs (Supplementary information, Figure S7G and S7H). Thus although CCL18 induces migration of naive CD4^+^ T cells into tumors, it does not cause them to differentiate into Tregs.

### PITPNM3 mediates CCL18-induced chemotaxis of naive CD4^+^ T cells

**Figure 5:**
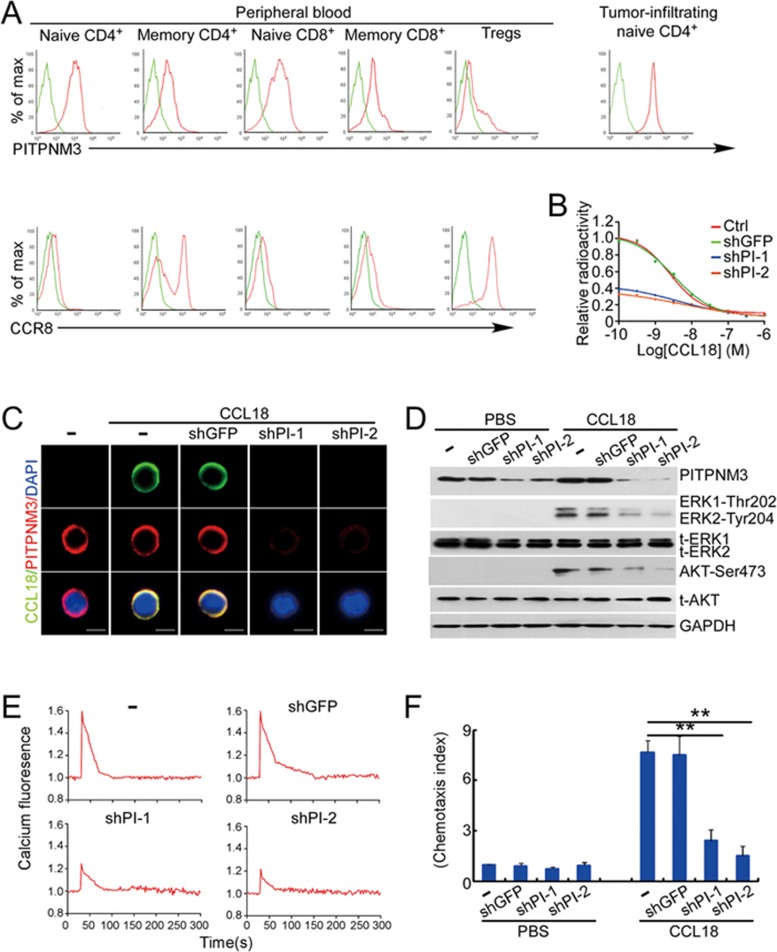
PITPNM3 is a CCL18 receptor on naive CD4^+^ T cells. **(A)** Representative flow cytometry staining for PITPNM3 and CCR8, potential CCL18 receptors, on gated PB T cell subsets and paired TI naive CD4^+^ T cells of a breast cancer patient. Cells were gated on CD3^+^CD45RA^+^CD45RO^−^CD25^−^CD4^+^/CD8^+^ for naive CD4^+^/CD8^+^ T cells, CD3^+^CD45RA^−^CD45RO^+^CD25^−^CD4^+^/CD8^+^ for memory CD4^+^/CD8^+^ T cells and CD3^+^CD4^+^CD25^+^ for Tregs). Quantitation of PITPNM3 and CCR8 expression on T cell subsets for eight breast cancer patients is provided in Supplementary information, Figure S8A. **(B-F)** Knockdown of *PITPNM3* in naive CD4^+^ T cells inhibits CCL18 binding, signaling and chemotaxis. **(B)** Binding of ^125^I-CCL18 to naive CD4^+^ T cells, knocked down or not for *PITPNM3* (shPI-1,2) in the presence of increasing concentrations of unlabeled CCL18. Shown are the representative assays for three independent experiments using PB T cells from three normal donors. **(C)** Representative fluorescence microscopy images of CCL18 binding to naive CD4^+^ T cells, knocked down or not for *PITPNM3*, stained for PITPNM3 and CCL18 3 h after adding CCL18. Scale bar, 5 μm. Shown are the representative images for three independent experiments using PB T cells from three normal donors. **(D)** Immunoblot of CCL18-treated naive CD4^+^ T cells, knocked down or not for *PITPNM3*, showing expression of PITPNM3 and phosphorylated/total (t-) Erk1/2 and Akt, relative to GAPDH as a loading control. Blots are representative of data for three donors. **(E)** Blunted [Ca^2+^]i mobilization in CCL18-treated naive CD4^+^ T cells knocked down for *PITPNM3*. Data are representative tracings for three donors. **(F)** Blunted chemotaxis of naive CD4^+^ T cells to CCL18 in a transwell assay. Data are shown as mean ± SEM. Chemotaxis indices for three independent experiments (^**^P < 0.01 by Student's *t*-test).

Although CCL18 preferentially attracts naive T cells, its receptors on these cells remain unidentified. PITPNM3 and CCR8 are reported CCL18 receptors on breast cancer cells and Th2 cells, respectively^21,26^. Moreover, a recent report showed that CCR8 is selectively overexpressed on TI Tregs, compared to normal tissue and PB Tregs^12^. Therefore, we examined the expression of PITPNM3 and CCR8 in T cell subsets from PB of breast cancer patients. CCR8 was only weakly expressed in naive T cell populations, but was more highly expressed on circulating Tregs and a subpopulation of memory CD4^+^ T cells (Figure 5A and Supplementary information, Figure S8A). In contrast, PITPNM3 was highly expressed by most naive CD4^+^ and CD8^+^ T cells (Figure 5A and Supplementary information, Figure S8A-S8D) in agreement with a previous report that CD4^+^ and CD8^+^ naive T cells respond to CCL18^22^, and more weakly on memory T cells. TI naive CD4^+^ T cells also had uniformly high PITPNM3 expression, but PB Tregs had low PITPNM3 expression. Based on these expression data, it is likely that PITPNM3, rather than CCR8, is the chemokine receptor on naive CD4^+^ T cells that responds to CCL18.

To investigate whether PITPNM3 mediates CCL18 binding on naive CD4^+^ T cells, we first assessed binding of ^125^I-CCL18 (Figure 5B). CCL18 specifically bound to naive CD4^+^ T cells with low nanomolar affinity (*Ki* = 6.5 nM, 5.8-7.3 nM, 95% confidence interval), and binding was competed by unlabeled CCL18. Moreover, knocking down *PITPNM3* by infecting naive CD4^+^ cells with a lentivirus expressing a PITPNM3 shRNA markedly inhibited ^125^I-CCL18 binding. Immunofluorescence microscopy using CCL18 and PITPNM3 antibodies showed that CCL18 co-localized with PITPNM3 on the surface of naive CD4^+^ T cells, which was abrogated by *PITPNM3* knockdown (Figure 5C). Furthermore, CCL18-induced Akt and Erk phosphorylation (Figure 5D), calcium influx (Figure 5E) and migration (Figure 5F) of naive CD4^+^ T cells were attenuated by *PITPNM3* knockdown. Thus, PITPNM3 is a CCL18 receptor on naive CD4^+^ T cells.

### *PITPNM3* knockdown in naive CD4^+^ T cells *in vivo* blocks their recruitment to tumors and Treg infiltration, reverses immunosuppression and inhibits tumor progression in humanized mice

**Figure 6:**
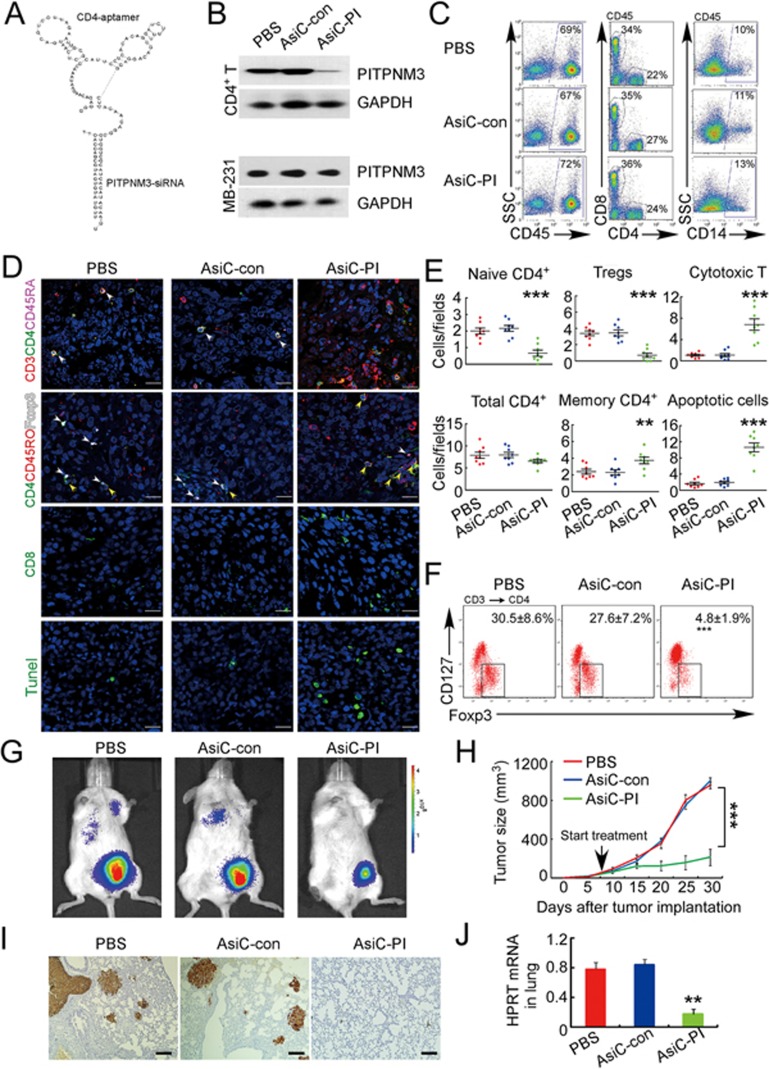
*In vivo* knockdown of *PITPNM3* in CD4^+^ T cells reverses immunosuppression and inhibits tumor progression in humanized mice. **(A)** Humanized mice bearing palpable MDA-MB-231 orthotopic xenografts were intraperitoneally injected daily for 14 days with PBS, 1 nmol CD4-aptamer-control siRNA (AsiC-con) or CD4-aptamer-siRNA targeting *PITPNM3* (sequence in **A**, AsiC-PI) to assess the role of PITPNM3 in TI Tregs, and other T cells and tumor control. Experimental schematic is provided in Supplementary information, Figure S9A. **(B)** Representative immunoblots showing selective knockdown of PITPNM3 protein in PB CD4^+^ T cells, but not tumor xenografts (*n* = 3). **(C)** *PITPNM3* knockdown did not affect the distribution of human CD45^+^ hematopoietic cells, CD4^+^ and CD8^+^ T cells, and CD14^+^ monocytes in the peripheral blood of humanized mice. Representative flow plots are shown (*n* = 3). **(D**, **E)** Effect of *PITPNM3* knockdown on TI naive CD4^+^, Tregs and CD8^+^ T cell numbers, and apoptosis by TUNEL assay in xenografts. **D** shows representative immunofluorescence microscopy images. Top row indicates CD4^+^ naive T cells by arrows; the second row indicates CD4^+^CD45RO^+^Foxp3^−^CD4^+^ memory T cells (yellow arrows) and Foxp3^+^ Tregs (white arrows). Scale bar, 50 μm. **E** shows number of cells of each subtype/high power field in eight mice (^**^P < 0.01, ^***^P < 0.001 compared to PBS group by Student's *t*-test). **(F)** Flow cytometry analysis of gated human CD3^+^CD4^+^ cells isolated from xenografts stained for CD127 and Foxp3. Representative flow plots in each group were shown. Numbers show the proportion of Tregs for eight mice per group (mean ± SEM; ^***^P < 0.001 compared to PBS group by Student's *t*-test). **(G)** Representative bioluminescence imaging of primary tumor and metastases in mice. **(H)** Primary tumor size (mean ± SEM) in each treatment group (8 mice per group; ^***^P < 0.001 by two-way ANOVA with Bonferroni multiple comparison tests). **(I)** Representative lung IHC images stained for human cytokeratin to identify human cancer cell metastases. Scale bar, 50 μm. **(J)** Quantification of metastatic lung tumors by qRT-PCR analysis of human *HPRT* mRNA relative to mouse 18S rRNA. Data are shown as mean ± SEM for eight mice per group (^**^P < 0.01 by Student's *t*-test).

Our data thus far suggest that breast TI Tregs develop from naive CD4^+^ T cells that are recruited to tumors when their PITPNM3 receptor recognizes CCL18 secreted by TAMs. To confirm the role of PITPNM3 in naive CD4^+^ T cell recruitment to breast tumors and elucidate the role of naive CD4^+^ T cell infiltration in Treg generation and cancer progression *in vivo*, we employed a humanized mouse tumor model. NOD/SCID/IL2rγ^null^ (NSG) mice were first transplanted with human fetal thymus and hematopoietic cord blood (CB) cells, and then inoculated in the mammary fat pad with MDA-MB-231 breast cancer cells (Supplementary information, Figure S9A). Gene expression can be efficiently and selectively knocked down in CD4^+^ cells *in vivo* by injecting CD4-aptamer-siRNAs chimeras (AsiC) (Figure 6A), RNAs composed of a CD4 aptamer linked to an siRNA^27^. We designed CD4-AsiCs to knock down *PITPNM3* (CD4-AsiC-PI) or a non-targeting control siRNA (CD4-AsiC-con). Once tumors became palpable, we injected these CD4-AsiCs intraperitoneally and verified by western blot and qRT-PCR that *PITPNM3* was selectively knocked down by the CD4-AsiC-PI, but not by the CD4-AsiC-con, in CD4^+^ T cells, but not in MDA-MB-231 cells (which also express *PITPNM3*) (Figure 6B and Supplementary information, Figure S9B). Injection of AsiC-PI did not alter human leukocyte reconstitution in the mice (Figure 6C). We next assessed by immunofluorescence microscopy and flow cytometry whether *PITPNM3* knockdown in CD4^+^ cells alters the numbers of types of CD4^+^ and CD8^+^ T cells in the xenografts (Figure 6D-6F, Supplementary information, Figure S9C). Mice that received CD4-AsiC-PI had significantly fewer naive CD4^+^ T cells and Tregs and more CD8^+^ T cells in the tumors compared to mice that received PBS or CD4-AsiC-con. The overall numbers of CD4^+^ cells in the tumor was not significantly different since there were more memory CD4^+^ T cells in the CD4-AsiC-PI-treated mouse tumors. Moreover, the number of human naive CD4^+^ T cells and Tregs were strongly correlated (*P*< 0.0001; Supplementary information, Figure S9D). The Tregs isolated from the tumors were functional, as they suppressed the proliferation of autologous PB CD8^+^ T cells stimulated with anti-CD3 and anti-CD28 (Supplementary information, Figure S9E). However, human Tregs were rare in the PB of the humanized mice (Supplementary information, Figure S9F), suggesting that Tregs were converted from naive CD4^+^ T cells in tumors. The reduction in TI Tregs and increase in TI CD8^+^ T cells were associated with more apoptosis in the tumor (Figure 6D and 6E). Mice treated with CD4-AsiC-PI also showed significantly smaller primary tumors and attenuated lung metastases (Figure 6G-6J). Thus knocking down *PITPNM3* in CD4^+^ cells reduced TI Tregs and enhanced TI CD8^+^ T cells, promoting anti-tumor immunity and tumor control. Of note, mice treated with CD4-AsiC-PI showed no significant signs of toxicity (Supplementary information, Table S5).

**Figure 7:**
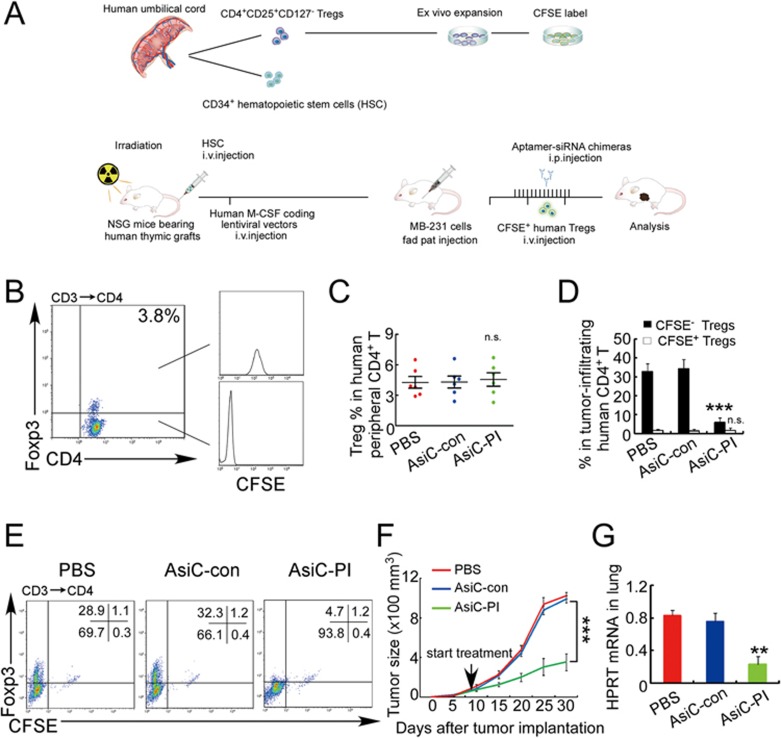
CD4-aptamer-siRNA targeting *PITPNM3* reduces TI Tregs and inhibits tumor progression in humanized mice with circulating human Tregs. Humanized mice, implanted with MDA-MB-231 tumors and concurrently injected intravenously with autologous Tregs, were intraperitoneally injected daily for 14 days after tumors became palpable with PBS, 1 nmol CD4-aptamer-control siRNA (AsiC-con) or CD4-aptamer-siRNA targeting *PITPNM3* to assess the role of PITPNM3 in TI Tregs, and other T cells and tumor control. Tregs were administered every 10 days after the initial injection and mice were sacrificed 30 days after tumor cell inoculation. **(A)** Experimental schematic. **(B**, **C)** Peripheral blood cells of humanized mice were stained for human CD3, CD4 and Foxp3, and analyzed by flow cytometry. A representative flow plot **(B)** and the percentage (mean ± SEM) of PB CD4^+^ cells that are CFSE^+^ Tregs in six mice per group **(C)** are shown. **(D**, **E)** Isolated cells from xenografts were stained for human CD3, CD4 and Foxp3. The percentage (mean ± SEM) of six mice per group **(D)** and representative flow plot **(E)** of FoxP3^+^ Tregs are shown. Most Tregs were CFSE^−^ (i.e., did not come from infused Tregs) and the number of TI Tregs was reduced by knocking down *PITPNM3* in CD4^+^ T cells (^***^P < 0.001 compared to the PBS group by Student's *t*-test). **(F)** Tumor size (mean ± SEM, *n* = 6 per group; ^***^P < 0.001 by two-way ANOVA with Bonferroni multiple comparison tests). **(G)** Lung metastases assessed by qRT-PCR analysis of human *HPRT* mRNA relative to mouse 18S rRNA in the lungs. Data are shown as mean ± SEM (*n* = 6 per group; ^**^P < 0.01 by Student's *t*-test). NS, not statistically significant by Student's *t*-test.

Because human PB Tregs were rare in the tumor-bearing humanized mice, to better mimic human breast cancer and determine whether PB Tregs infiltrate the tumors, we adoptively transferred autologous Tregs, expanded *ex vivo* from CB as previously described^28,29^, 6 weeks after the transplant of hematopoietic stem cells (HSCs) at the time of concomitant tumor implantation and every 10 days thereafter in the humanized tumor model described above. The transferred Tregs were labeled with CFSE (Figure 7A). After transfer, Foxp3^+^ Tregs accounted for about 4% of human PB CD4^+^ T cells (Figure 7B and 7C). Most of the PB Tregs were CFSE labeled, indicating that they were mainly adoptively transferred. When the tumors became palpable, the mice were injected intraperitoneally with PBS, CD4-AsiC-con or CD4-AsiC-PI. AsiC treatment did not affect the number of PB Tregs (Figure 7C). In the control mice given PBS or CD4-AsiC-con, Tregs accounted for about 30% of TI CD4^+^ T cells and most of these were CFSE^−^ (Figure 7D and 7E). The proportion of TI Tregs in the adoptively transferred mice was similar to that in the humanized mice that did not receive transferred Tregs (compare Figure 6F, Figure 7D-7E). These data, together with the paucity of CFSE-stained Tregs in the tumors from adoptively transferred mice, strongly suggest that TI Tregs were mainly derived from recruited naive CD4^+^ T cells, rather than from circulating Tregs. In fact, in the adoptively transferred tumor-bearing mice, knockdown of *PITPNM3* by CD4-AsiC-PI in CD4^+^ T cells markedly reduced CFSE^−^ TI Tregs, but had no effect on the small number of TI CFSE^+^ Tregs (Figure 7D and 7E). As before, CD4-AsiC-PI treatment dramatically reduced tumor growth and lung metastasis in the adoptively transferred tumor-bearing mice (Figure 7F and 7G). Together, these results suggest that Tregs are generated from naive CD4^+^ T cells responding via PITPNM3 to CCL18 produced in the tumor. They also suggest that interfering with PIPTNM3 or CCL18 could be used therapeutically to enhance anti-tumor immunity in breast cancer.

## Discussion

Although it is well documented that TI Tregs suppress the protective immune response to cancer, the origin of TI Tregs remains unclear^1,3,9^. In principle TI Tregs could arise from recruitment of circulating Tregs primed in the thymus or in peripheral LNs or from recruited naive or memory CD4^+^ T cells that are converted to Tregs in tumors^1,3,9^. Our results suggest that Tregs in human breast cancer are mainly converted from naive PB CD4^+^ T cells, recruited by TAM-derived CCL18. To build this case, we showed that CD4^+^ naive T cells are abundant in the stroma of extensively perfused samples — close to, but not within blood vessels — in most breast tumors compared to adjacent normal tissue; that their abundance correlates with TI Treg numbers; that the clonotypic TCR sequences of TI naive CD4^+^ T cells, rather than PB Tregs or TI memory T cells, are most similar to that of TI Tregs; and that the abundance of naive CD4^+^, but not CD8^+^, T cells in tumors is an independent negative prognostic factor for breast cancer. We then showed that naive CD4^+^ T cells from the PB of breast cancer patients differentiate *in vitro* into functional Tregs by exposure to autologous DCs and CM from their tumors. Although migration of naive T cells to peripheral lymphoid organs is mediated by CCL19 and CCL21^30^, migration of these cells to tumors is independent of these chemokines^31^. We implicated CCL18, previously identified as a breast cancer TAM-secreted chemokine^21,24^, in recruiting naive CD4^+^ T cells into breast tumors by binding to the chemokine receptor PITPNM3. Our evidence was that CCL18 was the only candidate chemokine overexpressed in human breast tumors relative to adjacent normal tissues; the abundance of TI naive CD4^+^ T cells correlated strongly with the abundance of CCL18^+^ TAMs; migration of PB naive CD4^+^ T cells into autologous tumor tissue slices correlated with the numbers of CCL18^+^ TAMs in the tumor; PITPNM3, but not CCR8 (another receptor for CCL18), was highly expressed by PB and TI naive CD4^+^ T cells compared to Tregs; CCL18 colocalized with PITPNM3 and its binding, signaling and chemotactic effect on naive CD4^+^ T cells was suppressed by *PITPNM3* knockdown. Most importantly, this model was validated in mouse models. Adoptively transferred human naive CD4^+^ T cells were recruited to orthotopic MDA-MB-231 triple-negative breast cancer tumors in NOD/scid mice and the recruitment was greatly enhanced by co-implantation of human macrophages and was dependent on CCL18, as it was suppressed by injection of anti-CCL18 (Figure 4E). We also tested this model in tumor-bearing mice transplanted with human HSCs and fetal thymus that develop a human immune system (Figures 6 and 7). Systemic and selective knockdown of *PITPNM3* in CD4^+^ T cells achieved by intraperitoneal injection of CD4-AsiCs blocked recruitment of TI naive CD4^+^ T cells and development of TI Tregs, caused expansion of TI CD8^+^ T cells and increased intratumoral apoptosis, and strongly suppressed primary tumor growth and metastases.

Naive T cells were previously thought to traffic exclusively to lymphoid organs. Growing evidence suggests that this viewpoint is too simplistic^32,33^. First, it is well documented that naive T cells can enter non-lymphoid organs that contain tertiary lymphoid structures^33,34,35^. More recent reports also indicate that naive T cells can enter non-lymphoid organs in the absence of lymphoid structures. Naive T cells have been found in many non-lymphoid organs, especially in the lungs, as part of normal migratory pathways in normal mice^36,37^, and in inflamed tissues^38^ and in tumors^31,39^ of mice. Moreover, the presence of naive CD4^+^ T cells has been previously noted in human breast cancer^40^, although their role and significance were not previously explored.

Our findings in human samples are in agreement with previous reports that Tregs in murine breast cancers are derived from resting CD4^+^ T cells^41,42^. Previous studies in mouse or human ovarian cancers suggested that TI Tregs could be recruited from the circulation to tumors by CCL22 and CCL28. However, CCL22 and CCL28 expression is not increased in human breast cancers compared to normal breast tissue, making them unlikely Treg recruiters to human breast cancer. Different cytokine expression profiles in different cancer types may lead to distinct sources of TI Tregs. Future research will need to determine to which other types of tumors our model of TI Treg development from recruited naive T cells applies.

A recent study found that the TCR repertoire of TI Tregs showed low overlap with that of normal breast tissue or PB Tregs or TI effector (CD25^−^) T cells^12^. Their results, in agreement with ours, suggest that TI Tregs do not arise from either recruitment of Tregs from the periphery or from conversion of activated conventional T cells within the tumor, in agreement with our model. However, that study did not compare the repertoires of Tregs with PB or TI naive CD4^+^ T cells and hence did not address whether Tregs might arise from recruited PB naive CD4^+^ T cells. Our TCR sequence analysis also showed that the TCRs of TI memory CD4^+^ T cells overlapped with TCR sequences of PB and LN effector/memory CD4^+^ T cells, and LN naive CD4^+^ T cells. Our results, therefore, suggest that tumor-reactive effector CD4^+^ T cells are primed in LNs. Therefore, generation of anti-tumor effector T cells and TI Tregs involve different chemokine responses and priming locations. It is possible to take advantage of these differences to reduce Treg progenitors without interfering with, or even (as our data suggest) boosting anti-tumor immunity.

Although CCR8 and PITPNM3 were identified as potential CCL18 receptors on other cells^21,26^, the CCL18 receptor in naive T cells was not known. In agreement with a previous report^43^, we did not find that naive T cells express CCR8. In contrast, PITPNM3 is highly expressed in naive CD4^+^ T cells and mediates their CCL18 binding and signaling. However, a large fraction of Tregs and memory T cells express CCR8, which might recruit them to breast tumors (Figure 5A). Besides chemokine receptors, other regulatory factors mediate lymphocyte chemotaxis to chemokine gradients, including regulator of G-protein signaling^44^. Overexpression of RGS1, RGS9 and RGS16 in Tregs, compared to naive CD4^+^ T cells, accounts for their distinct response to CCL19 and CCL21 in mice^45^. It is worth investigating whether differential expression of chemokine receptors together with RGS or other regulatory factors affects the chemotaxis of Tregs, and naive and effector CD4^+^ T cells to CCL18 secreted in tumors.

A previous study showed that TI Tregs rapidly recover after they are depleted from tumor-bearing mice^42^. Our finding here suggests that inhibiting the *in situ* generation of TI Tregs might be a better strategy for cancer immunotherapy than depleting TI Tregs. Depletion of TI Tregs also carries the risk of systemic autoimmunity, unless TI Tregs can be selectively depleted without eliminating tissue Tregs. A recent study found that CCR8 is highly expressed by TI Tregs and not by PB Tregs, and suggested the therapeutic use of anti-CCR8 to selectively deplete TI Tregs^12^. However, Tregs in normal breast tissue also highly express CCR8 (about half as much as in tumors), suggesting that normal tissue Tregs might also be depleted. Experiments in immunocompetent mice, such as in the tumor-bearing humanized mouse model we used here or in genetically engineered mouse models of cancer, might determine whether depleting CCR8^+^ cells is safe and effective, and could be used to compare different approaches to control Treg suppression of tumor immunity.

In this study we showed that inhibiting naive CD4^+^ T cell recruitment by knocking down *PITPNM3* in tumor-bearing humanized mice could reduce TI Tregs, restore immune killing of tumors and suppress tumor growth and metastases, confirming the role of PITNM3 recognition of CCL18 in TI Treg biogenesis. To achieve *in vivo* knockdown, we intraperitoneally injected CD4-AsiCs targeting *PITPNM3* and showed that they selectively knock down gene expression in CD4^+^ T cells, but not in tumors. Previously topical administration of CD4-AsiCs was used to knock down genes in human CD4^+^ T cells, without activating the cells or inducing innate immunity, in the genital tract of humanized mice to prevent HIV transmission^27^. Our unpublished data also show that subcutaneous injection of CD4-AsiCs leads to systemic and selective *in vivo* gene knockdown in CD4^+^ cells. Thus CD4-AsiCs provide a valuable tool to interrogate the role of individual gene products in CD4 cells in immune protection and pathology in general and in tumor immunity in particular that could potentially be developed for therapeutic use.

Inhibiting naive T cell recruitment into tumors by interfering with the CCL18-PITPNM3 interaction is a previously unappreciated potential strategy for tumor immunotherapy. It is dually beneficial — it not only inhibits the numbers of immunosuppressive Tregs in the tumor, but also increases the numbers of intratumoral protective effector CD8^+^ T cells, presumably because they are not suppressed from proliferating in response to the tumor. It is worth noting that neutralizing CCL18 has no appreciable effect on tumor growth in NOD/scid mice bearing orthotopic breast tumors, which are not “humanized”, i.e., reconstituted with human immune cells (Supplementary information, Figure S5B). The difference between that model and the humanized mice indicates that tumor suppression was mediated by enhancing specific anti-tumor immunity. In the future it will be worth examining in tumor-bearing humanized mice whether blocking CCL18 or PITPNM3 also leads to enhanced anti-tumor immunity and tumor suppression, and to compare it with anti-CCR8 and with CD4-AsiCs against *PITPNM3* or *CCR8*. In this study, CD4-AsiCs against *PITPNM3* did not affect PB T cell subset numbers or lead to any apparent toxicity in humanized mice. The safety and lack of autoimmunity secondary to CD4-AsiC knockdown of *PITPNM3* or antibody inhibition of CCL18, PITPNM3 or CCR8 will need to be examined carefully to evaluate potential therapeutic applications. Nevertheless, our study suggests that blocking naive CD4^+^ T cell recruitment and conversion to Tregs in tumors could be an attractive new immune therapeutic strategy for cancer.

## Materials and Methods

### Primary cell isolation from breast cancer and blood

Primary cells were obtained from blood and tissues as previously described^4,24^. Briefly, tumor tissues and LN obtained from ipsilateral axillary LN dissection were exhaustively perfused with PBS, minced into small (1-2 mm in diameter) pieces and digested with 5% FBS DMEM containing 2 mg/ml collagenase I and 2 mg/ml hyaluronidase (Sigma) at 37 ^o^C for 2 h and 0.5 h, respectively. The cells were sequentially filtered through 500 μm mesh, 100 and 70 μm cell strainers, and then centrifuged in a Beckman Allegra X-15R centrifuge at 2 500 rpm for 20 min with 1 ml cell suspension above 5 ml 45% Percoll (GE Healthcare) in the middle and 5 ml 60% Percoll at the bottom in a 15-ml tube. Primary tumor cells were collected from the cell layer in the interface above 45% Percoll and further purified by Cancer Cell Isolation Kit (Panomics). Mononuclear cells were collected from the cell layer at the interface between 45% and 60% Percoll. Mononuclear cells from LN or 50 ml PB were also isolated by Ficoll density gradient centrifugation. Naive CD4^+^ T cells, Tregs, memory CD4^+^ T cells, CD8^+^ T cells, pDCs or myeloid DCs were purified using the Naive CD4^+^ T Cell Isolation kit (130-094-131, Miltenyi), CD4^+^CD25^+^CD127^−^ Regulatory T Cell Isolation Kit (130-094-775, Miltenyi), Memory CD4^+^ T Cell Isolation Kit (130-091-893, Miltenyi), CD8^+^ T Cell Isolation kit (130-094-156, Miltenyi), Diamond Plasmacytoid Dendritic Cell Isolation Kit (130-097-240, Miltenyi) and CD1c (BDCA-1)^+^ Dendritic Cell Isolation Kit (130-090-506, Miltenyi), respectively. The Tregs in naive/memory CD4^+^ T cells were depleted using CD25 microbeads (130-092-983, Miltenyi). Cell populations were confirmed to be > 90% pure by FACS.

### TCR sequencing

For all T cell subsets, equal numbers of purified cells from each patient (∼5-20 × 10^4^) were used for TCR sequencing. TCR-β/α variable region full-length sequencing was performed as previously described^50,51^ with slight modifications. Total RNA was extracted and purified from T cell samples using ReliaPrep RNA Cell Miniprep System (Z6011, Promega), and was reverse transcribed into first-strand 5′RACE-ready cDNA by using SMARTer RACE 5′/3′Kit (634859, TaKaRa) to add universal adapters at the 5′-end of the RNA and 3′-end of the cDNA. First round 5′RACE PCR was performed using Advantage 2 Polymerase (Clontech) with 10× Universal Primer Mix (UPM, Clontech) and forward primer, and self-designed TCR constant region first round reverse primers (TCR: 5′-GGTGTCTTCTGGAATAATGCTGT-3′, TCR: 5′-GCTCAGGCAGTATCTGGAGTCATTG-3′). Amplification was performed on a Applied Biosystems Veriti Thermal Cycler using the following conditions: 1 min at 95 °C; 5 cycles of 20 s at 95 °C and 30 s at 72 °C; 5 cycles of 20 s at 95 °C, 30 s at 70 °C and 30 s at 72 °C; 25 cycles of 20 s at 95 °C, 30 s at 60 °C and 30 s at 72 °C; and 5 min at 72 °C. Agarose gel-purified PCR products were then amplified (Semi-nested PCR) with Nested Universal Primer A forward primer (Clontech) and self-designed second round reverse primers (TCRα constant region: 5′-CAGGGTCAGGGTTCTGGAT-3′, TCRβ: 5′-CACAGCGACCTCGGGTGGGAA-3′). Amplification conditions were: 1 min at 95 °C; 30 cycles of 20 s at 95 °C, 30 s at 60 °C and 30 s at 72 °C; and 5 min at 72 °C. Agarose gel-purified libraries were bar coded with multiplex adaptors and sequenced on Illumina MiSeq. The sequences of sample barcodes employed for multiplexing are given in Supplementary information, Table S6. For TRBV12-4/TRBJ1-2 sequencing, we amplified clonotypes using adaptor-ligated barcode primers listed in Supplementary information, Table S6. Agarose gel-purified PCR products were sequenced using Ion Personal Genome Machine (PGM) system (ThermoFisher). The Sequence Read Archive (SRA) database (www.ncbi.nlm.nih.gov/sra/) accession number for the TCR sequences reported in this paper is SRP065925. The similarity between the different TCR repertoires was calculated by the Morisita-Horn similarity index, which takes into account not only the number of shared sequences between two repertoires but also the contribution of those shared sequences to each repertoire^16^.

### Patients and tissue samples

Primary breast carcinomas were obtained from 481 patients at Sun Yat-Sen Memorial Hospital, Sun Yat-Sen University (Guangzhou, China) and 145 patients at the First Affiliated Hospital, Shantou University Medical College (Shantou, China). Benign breast tissue samples from 59 cases of cystic fibrosis with or without atypical epithelial hyperplasia and tumor samples from 39 cases of DCIS were collected at Sun Yat-Sen Memorial Hospital. All samples were collected with informed consent with the approval of the Internal Review and Ethics Boards of the indicated hospitals.

### IHC and immunofluorescent staining of tissue

Antigen retrieval was performed by incubating slides in a pressure cooker for 5 min in 0.01 M citrate buffer (pH 6.0), followed by treatment with 3% hydrogen peroxide for 5 min. Slides were incubated overnight at 4 °C with the following antibodies: CD3 (Cat# M7254, mouse anti-human, DAKO; 1:100 or Cat# sc-1128, goat anti-human, Santa Cruz; 1:50), CD45RA (Cat# M0754, mouse anti-human, DAKO; 1:50), CD4 (Cat# PA5-11582, rabbit anti-human, Thermo Fisher Scientific; 1:100 or Cat# ab34276, rat anti-human, Abcam; 1:20), Foxp3 (Cat# ab20034, mouse anti-human, Abcam; 1:50 or Cat# ab54502, rabbit anti-human, Abcam; 1:50), CD8 (Cat# MA5-14548, rabbit anti-human, Thermo Fisher Scientific; 1:200), CD45RO (Cat# M0742, mouse anti-human, DAKO; 1:200), CD62L (Cat# ab135792, rabbit anti-human, Abcam; 1:50), CD20 (Cat# ab78237, rabbit anti-human, Abcam; 1:100), CD68 (Cat# sc-7083, goat anti-human, Santa Cruz; 1:100), CCL18 (Cat# MAB394, mouse anti-human, R&D; 20 μg/ml) or human cytokeratin (Cat# ab756, mouse anti-human, Abcam; 1:50). IHC was performed using the double stain kit (Dako) according to the manufacturer's instructions. For immunofluorescence, specimens were incubated with Alexa Fluor secondary antibodies (Thermo Fisher, China). For negative control, isotype-matched antibodies were used. Immunostaining was evaluated using TMAJ Image (http://tmaj.pathology.jhmi.edu) computerized image analysis. The accuracy of automated measurements was confirmed by independent evaluation by two pathologists (YZ and NO). Cells stained with the indicated antibodies were counted at 400× magnification in at least 10 fields per section.

### Co-culture of naive CD4^+^ T cells and DCs

2 × 10^3^-1 × 10^4^ TI DCs and PB DCs, and autologous PB CD4^+^ naive T cells were isolated as described above and co-cultured (1:10-20 DCs:T cells)^19^ in growth medium (RPMI 1640 with 10% FBS and 25 U/ml IL-2) with or without 30% CM from autologous tumor slices or adjacent normal tissue slices for 9 days. Non-adherent T cells were then harvested for further experiments.

### Suppression assay

DC-primed Tregs were harvested, purified using CD25 (130-092-983, Miltenyi) and CD127 (130-094-945, Miltenyi) microbeads, and evaluated for their suppressive capacity as previously described^46,47^. Briefly, immature myeloid DCs were produced by culturing autologous monocytes isolated from PB in DMEM containing 25 ng/ml GM-CSF, 5 ng/ml IL-4 (PeproTech) and 10% heat-inactivated autologous serum for 6 days. The cultures were replaced with fresh medium and cytokines every 3 days, and cell differentiation was monitored by light microscopy. Myeloid DCs were matured by incubation with 100 ng/ml LPS and 500 U/ml IFN-γ (PeproTech) for 48 h and then pulsed for 24 h with lysates (200 μg protein/1 × 10^6^ cells/ml) from isolated autologous tumor cells lysed by 5 freeze/thaw cycles. Autologous CD8^+^ T cells isolated from PB were labeled with 0.5 μM CFSE (Invitrogen) for 15 min at RT and incubated with mature myeloid DCs (5:1) in the presence or absence of DC-primed Tregs at the indicated ratios in RPMI 1640 medium supplemented with 5 μg/ml IL-12, 25 mmol/l HEPES, 4 mmol/l ℒ-glutamine, 25 μmol/l 2-mercaptoethanol and 10% heat-inactivated autologous serum. Proliferation of CD8^+^ T cells was measured by CFSE staining and flow cytometry after 5 days.

### Cytotoxicity assay

The Live/Dead Cell Mediated Cytotoxicity Kit (Molecular Probes) was used as previously described^48^. Briefly, primary breast cancer cells were stained with 3,39-dioctadecyloxacarbocyanine (DiOC18) for 15 min at RT. Tumor-specific CD8^+^ T cells (2 × 10^4^), generated by incubation with tumor lysate-pulsed DCs as described above, were harvested using CD8 microbeads (130-045-201, Miltenyi) and added to cancer cells stained with propidium iodide (3.75 mM solution, 1:500 final dilution) at an effector-to-target ratio of 10:1. After 18 h, cells were harvested and analyzed by flow cytometry.

### *Ex vivo* tumor slice migration assay

*Ex vivo* tumor slice migration assay was performed as previously described^23^. Briefly, freshly resected samples were embedded in 5% agarose (type VII-A; Sigma-Aldrich), 400 μm slices were cut with a vibratome (VT 1000S; Leica), overlaid with 2 × 10^5^ autologous naive CD4^+^ T cells isolated from PB and labeled with CFSE, and incubated in 24-well plates containing RPMI 1640 plus 10% autologous serum for 3 h. The slices were then rinsed, fixed and stained with anti-CCL18 (Cat# MAB394, R&D). Cells were counted in at least 10 fields of view per section at 400× magnification.

### *In vivo* migration assay

*In vivo* migration was assayed as previously described^30^ with slight modifications. Healthy donor PB naive CD4^+^ T cells (1 × 10^6^) were labeled with CFSE and injected via tail vein into female NOD/scid mice, bearing tumors formed 14 days earlier by mammary fat pad injection of 2 × 10^6^ MDA-MB-231 cells (American Type Culture Collection), with or without 2 × 10^6^ autologous macrophages. For inhibition experiments, 10 μg per mouse of isotype control (Cat# ab172569, Abcam) or anti-CCL18 (Cat# ab9849, Abcam) was added to naive CD4^+^ T cells before injection. For recombinant protein treatment, rhCCL18 (0.1 mg/kg, PeproTech, Rocky Hill, NJ, USA) was injected intratumorally into MDA-MB-231 xenografts 1 h before T cells were injected. Xenografts were harvested 24 h after T cell injection and sectioned. CFSE^+^ cells were counted in at least 10 fields per section at 400× magnification.

### Flow cytometry

Cells were stained with CD3 FITC (Cat# 11-0039), CD3 eFluor 450 (Cat# 48-0038), CD4 PE-Cyanine7 (Cat# 25-0049), CD4 PerCP-Cyanine5.5 (Cat# 560650, BD Pharmingen), Foxp3 APC (Cat# 17-4777), Foxp3 PE (Cat# 12-4776), CD45RA PE (Cat# 130-092-248, Miltenyi), CD45RA APC (Cat# 130-092-249, Miltenyi), CD62L APC (Cat# 17-0629), CD25 PE (Cat# 130-091-024, Miltenyi), CD25 APC-H7 (Cat# 560225, BD Pharmingen), CD8 PE-Cyanine7 (Cat# 25-0088), Perforin PE (Cat# 12-9994), granzyme B PE (Cat# 12-8899), CCR8 APC (Cat# FAB1429A, R&D System), CD4 FITC (Cat# 11-0049), CD45 APC (Cat# 17-9459), CD14 PE (Cat# 12-0149), CD45RO PE (Cat# 12-0457), CD45RO eFluor 450 (Cat# 48-0457), CD127 APC (Cat# 17-1278). PITPNM3 antibody (Cat# NBP1-31070, Novus) was labeled with APC by Abcam APC Conjugation Kit (ab201807, Abcam) according to the manufacturer's instructions. For the intracellular stain, cells were pretreated with Intracellular Fixation and Permeabilization kit (Cat# 88-8824) according to the manufacturer's instructions. All the reagents were from eBioscience unless indicated otherwise. Cells were subsequently analyzed by multicolor flow cytometry (Gallios, Beckman Coulter, China).

### qRT-PCR

qRT-PCR was performed with a LightCycler 480 instrument (Roche Diagnostics, Switzerland), using the SYBR Premix Ex Taq TM (TaKaRa, Japan) according to the manufacturer's instruction. For absolute quantitative PCR, we determine the absolute level of PITPNM3 mRNAs as previously reported^49^. Briefly, we constructed standard sample using the same primers of PITPNM3 as for real-time PCR, the amplified DNA fragments were purified and cloned into pEASY-T5 Zero cloning vector (CT501, TransGen Biotech, China), and transformed into DH5α competent cells (TaKaRa). Finally, plasmid DNA was extracted using Plasmid Mini Kit (D6943, Omega Bio-Tek, Doraville, USA). Equivalent molecules per cell were extrapolated from the standard curve and threshold cycle (*C*_t_) values, based on the assumption that total RNA per cell is 20 pg. The primer sequences are listed in Supplementary information, Table S6.

### Muc1-specific CTL assay

A Muc1 peptide (LLLLTVLTV) and a HER-2-/neu-derived peptide (KIFGSLAFL) were synthesized by Abgent Biotech Ltd (Suzhou, China)^50^. DCs from HLA-A2^+^ patients (determined by flow cytometry analysis for HLA-A2, Cat# 343303, Biolegend) with tumors expressing Muc1 (determined by IHC staining for Muc1, Cat# ab109185, Abcam) were pulsed with 50 μg/ml synthetic peptide for 8 h, washed, and incubated with autologous CD8^+^ T cells (1:5) isolated from PB in the presence or absence of DC-primed Tregs at various ratios in RPMI 1640 medium supplemented with 5 μg/ml IL-12, 25 mmol/l HEPES, 4 mmol/l ℒ-glutamine, 25 μmol/l 2-mercaptoethanol and 10% heat-inactivated autologous serum. Antigen specificity of tumor cell lysis was further determined in a cold target inhibition assay by analyzing the capacity of unlabeled T2 cells (American Type Culture Collection) coated with the Muc1 peptide or the HER-2/neu-derived peptide to block lysis of tumor cells at a ratio of 20:1 (inhibitor to target ratio).

### Primary T cell transduction

Primary T cell transduction was performed as described before^51^. Briefly, primary T cells were isolated from PB of healthy donors as described above and cultured in growth medium (RPMI 1640 with 10% fetal bovine serum and 25 U/ml IL-2). After 12 h, PITPNM3 shRNAs with previously proved potency and specificity were delivered^24^ to T cells by 2-4 × 10^7^ lentiviral particles (multiplicity of infection (MOI) of 10-20, Genepharma, Shanghai, China) supplemented with 8 μg/ml Polybrene (Sigma) for 8-10 h, then the cell suspension was spun and replaced with the growth medium. The transduction was repeated in two consecutive days and T cells were collected for later experiments.

### Cell immunofluorescent staining

Naive CD4^+^ T cells isolated from PB of healthy donors were incubated with primary antibodies against CCL18 (Cat# MAB394, mouse anti-human, R&D; 20 μg/ml) or PITPNM3 (Cat# NBP1-31070, rabbit anti-human, Novus; 1:100), followed by incubation with Alexa Fluor 488 donkey anti-mouse IgG (H+L) and Alexa Fluor 555 goat anti-rabbit IgG (H+L) (Thermo Fisher). For confocal microscopy, the cells on cover slips were counterstained with DAPI and imaged using a confocal laser-scanning microscope (Carl Zeiss) with a core data acquisition system (Applied Precision).

### Binding assays

Competition experiments were performed by using ^125^I-labeled CCL18 and the indicated concentrations of unlabeled chemokines as previously described^21^. Briefly, 2 nM ^125^I-labeled CCL18 was incubated with 2 × 10^5^ naive CD4^+^ T cells isolated from PB of healthy donors resuspended in 100 μl binding buffer (50 nM HEPES, pH 7.2, 1 mM CaCl_2_, 5 mM MgCl_2_, 0.5% BSA) in the presence of the indicated concentrations of unlabeled CCL18. After incubation at room temperature (RT) for 1 h, the cells were pelleted through a PBS cushion with 10% sucrose for 1 min at 10 000× *g*. The supernatant was removed and the radioactivity associated with cell pellets was measured using a liquid scintillation counter (CliniGamma, Pharmacia). Each data point was determined in triplicate. Binding data were analyzed with Prism computer program by GraphPad (San Diego, CA, USA).

### Western blot

Protein extracts were resolved through 8%-15% SDS-PAGE, transferred to PVDF membranes, and probed with antibodies against PITPNM3 (Cat# NBP1-31070, Novus), Akt (Cat# 4685, CST), phospho-Akt (Cat# 4060, CST), ERK (Cat# 4695, CST), phospho-ERK (Cat# 4370, CST), GAPDH (Cat# HRP-60004, Proteintech). Peroxidase-conjugated anti-mouse or -rabbit antibody (CST) was used as secondary antibody and the antigen-antibody reaction was visualized by enhanced chemiluminescence assay (ECL, Thermo).

### Calcium mobilization assay

Naive CD4^+^ T cells isolated from PB of healthy donors were suspended at 3 × 10^6^ cells/ml in Hank's balanced salt solution (HBSS) containing 1 mg/ml of bovine serum albumin (BSA) and 10 mM HEPES, pH 7.4 (HBSS-BSA) and incubated with 1 mM Fura-2-AM (Dojindo, Kumamoto, Japan) at RT for 30 min in the dark. After washing twice with HBSS-BSA, cells were suspended in HBSS-BSA at 2.5 × 10^6^ cells/ml. 2 ml of the cell suspension in a quartz cuvette was placed in a luminescence spectrometer (RF-5000, Shimadzu, Kyoto, Japan) and fluorescence was monitored at an emission wavelength of 510 nm, and excitation wavelengths of 340 and 380 nm every 20 ms. Calibration of fluorescence in terms of [Ca^2+^]i was calculated from the ratio 340/380 excitation fluorescent values.

### Chemotaxis assays

For chemotaxis assays, the indicated T cells isolated from PB of healthy donors transmigrated across 5-mm transwell filters (Costar, Cambridge, MA, USA) for 6 h to medium with or without 20 ng/ml rhCCL18 (PeproTech) in the bottom chamber, and were enumerated by flow cytometry^52^. Transwell assays were performed in triplicate and repeated using cells from a minimum of three different donors.

### Humanized mouse experiments

Animal work was approved by the Institutional Review Boards and Animal Care and Use Committees of Sun Yat-Sen University and the University of Hong Kong. Humanized mice were generated from NOD/SCID/IL2rγ^null^ (NSG) mice (Jackson Laboratories) as previously described^27,53^. Fresh human CB and fetal thymi were obtained from Sun Yat-Sen Memorial Hospital, according to guidelines approved by the hospital Ethics Boards and Clinical Research Committee. CD34^+^ HSCs were isolated to > 95% purity using two rounds of selection with the direct CD34 Progenitor Cell Isolation Kit (Miltenyi Biotec) from CB mononuclear cells isolated by Ficoll-Hypaque density gradient centrifugation. Briefly, 3-4-week female NSG mice bearing human surgical thymic grafts were subjected to 200 cGy total body irradiation 12 h before tail vein injection with 2 × 10^5^ HSCs in 0.2 ml of medium^54^. Mice were injected with 0.1 ml of human M-CSF-encoding lentiviral vectors (5-10 × 10^8^ total TU/ml) via tail vein 1 week after transplantation^24^. Six weeks after HSC transplantation, 2 × 10^6^ MDA-MB-231 cells, stably expressing luciferase, were injected into the mammary fat pad. For AsiC treatment, mice were injected intraperitoneally with PBS, CD4-AsiC-con (1 nmol) or CD4-AsiC-PI (1 nmol) daily for 14 days^55^ after xenografts were palpable using AsiCs from TriLink BioTechnologies. Tumor growth was evaluated by monitoring tumor volume (TV = length × width^2^ × 0.5) every 5 days. Whole-body tumor burden of animals bearing xenografts was assessed after luciferin injection using the IVIS Lumina Imaging System (Xenogen). Animals were sacrificed when xenografts reached 1.5 cm in diameter. The blood, tumor xenografts and lungs of the sacrificed mice were harvested for further investigation. Total RNA was extracted from lungs for qRT-PCR analysis of human HPRT mRNA expression. For some experiments, autologous CD4^+^CD25^+^CD127^−^ Tregs were also isolated from CB by magnetic bead isolation (Miltenyi Biotec) and cryopreserved. Four weeks after HSC transplantation, Tregs were thawed and expanded *ex vivo* with 1 000 U/ml of rhIL-2, 5 ng/ml rhTGF-β (PeproTech) and αCD3/αCD28 beads (Invitrogen) in a 1:2 cell to bead ratio for two 7-day rounds of stimulation. The expanded Tregs (1 × 10^5^) were labeled with CFSE for 15 min at RT and intravenously injected into humanized mice every 10 days as indicated.

### Statistics

All statistical analyses were performed using SPSS for Windows version 13.0 (SPSS, Chicago, IL, USA). Pearson correlation and regression analysis was used to assess the relationship between naive CD4^+^ T cell number, Treg cell number and CCL18^+^ cell number in human breast tissue. X-Tile statistical software was used to group clinical samples. Kaplan-Meier survival curves were plotted and log-rank test was done. All *in vitro* experiments were performed in triplicate in least three independent experiments. *P* < 0.05 was considered statistically significant.

## Author Contributions

SS, J Lieberman, QL and ES conceived and designed the experiments. SS, J Liao, J Liu, DH, CH, FC and LY performed the experiments. WW, LL, XC, HY and FS provided the clinical samples and facilities. SS, J Liao, DH, J Liu, FC, CH, YZ and NO analyzed the data. SS, J Liberman, QL and ES wrote the paper.

## Competing Financial Interests

The authors declare no competing financial interests.

## Supplementary Information

Supplementary information, Figure S1 The purity of isolated T cell populations

Supplementary information, Figure S2 TCR-β/α gene usage of isolated T cell populations

Supplementary information, Figure S3 CD4^+^ CD45RA^+^ T cells in breast cancer are associated with Treg enrichment.

Supplementary information, Figure S4 CD4^+^ CD62L^+^ T cells in breast cancer are associated with poor patient prognosis.

Supplementary information, Figure S5 Naïve CD4^+^ T cells are converted to functional Tregs by tumor-infiltrating myeloid DCs and tumor conditioned medium.

Supplementary information, Figure S6 Tregs Converted from Naïve CD4^+^ T Cells Suppress Tumor-associated Antigen-Specific CTLs

Supplementary information, Figure S7 Naïve CD4^+^ T cells are recruited to breast tumors by TAM-secreted CCL18.

Supplementary information, Figure S8 PITPNM3 is expressed in naïve CD4^+^ T cells.

Supplementary information, Figure S9 CD4 aptamir-PITPNM3 siRNA reverses immunosuppression and inhibits tumor progression in humanized mice.

Supplementary information, Table S1 Correlation between tumor infiltrating naïve CD4^+^ T cells and clinicopathological parameters

Supplementary information, Table S2 Cox regression analysis of DFS in entire cohort (n=626)

Supplementary information, Table S3 Correlation of CD4^+^ naïve T cell associated gene expression and clinical features in breast cancer in the Oncomine online database

Supplementary information, Table S4 T cell chemotactic cytokine expression in breast cancer in the online databases

Supplementary information, Table S5 Cardiac and liver functions in humanized mice injected with aptamer-siRNA chimeras

Supplementary information, Table S6 The PCR primer sequences
