# Supplementary material for: Blocking the recruitment of naive CD4+ T cells reverses immunosuppression in breast cancer
Source: Cell Res. 2017 Mar 14;27(4):461–82. doi: 10.1038/cr.2017.34 (PMC5385617; doi:10.1038/cr.2017.34)
Supplement: Supplementary information, Figure S1 — The purity of isolated T cell populations [file cr201734x1.pdf]

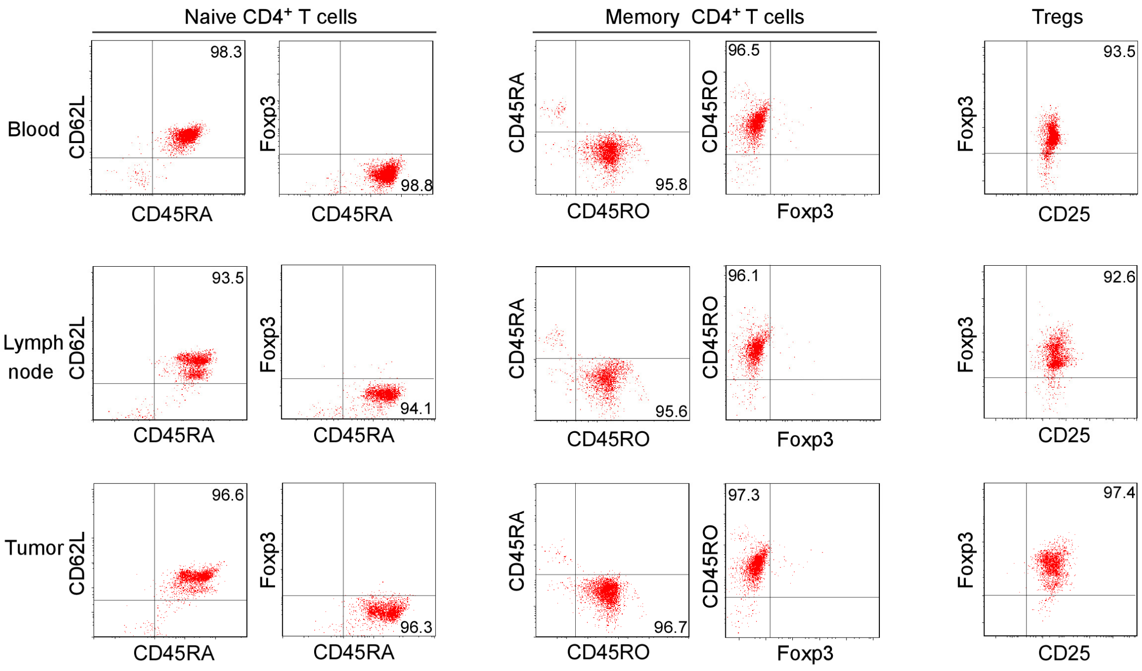

Supplementary Figure 1. The purity of isolated T cell populations

Tregs, naïve $CD4^+$ T cells and memory $CD4^+$ T cells were isolated from peripheral blood, lymph nodes and primary tumors of breast cancer patients , stained for indicated markers and analyzed by flow cytometry. The purity of isolated T cell subsets was more than 90%. Representative flow plots are shown.
