# Supplementary material for: Blocking the recruitment of naive CD4+ T cells reverses immunosuppression in breast cancer
Source: Cell Res. 2017 Mar 14;27(4):461–82. doi: 10.1038/cr.2017.34 (PMC5385617; doi:10.1038/cr.2017.34)
Supplement: Supplementary information, Figure S2 — TCR-β/α gene usage of isolated T cell populations [file cr201734x2.pdf]

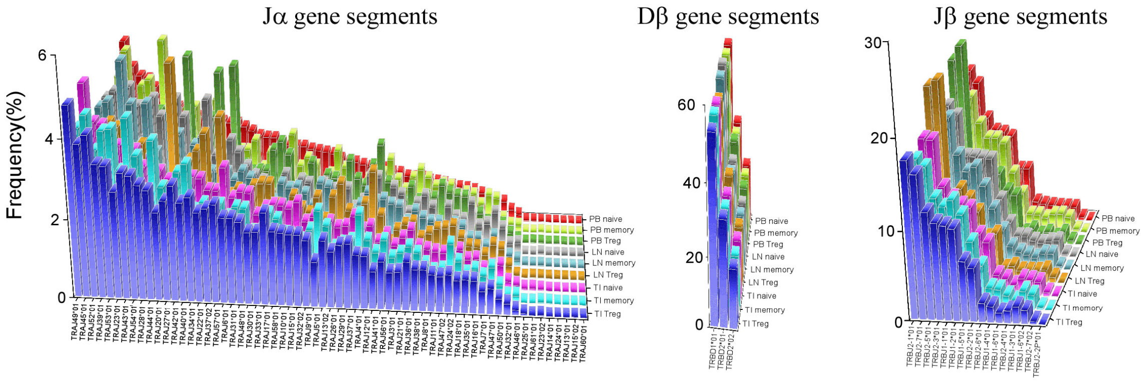


**Supplementary Figure 2. TCR-β/α gene usage of isolated T cell populations**

Full length TCR-β/α variables were amplified by 5′ RACE PCR from isolated T cells, and their TCR-β/α repertoires were compared by high throughput sequencing. Frequencies of Jα/β and Dβ gene usage in the groups of isolated T cells. (Jα/β and Dβ genes were ordered based on decreasing frequency in PB naïve $CD4^+$ T cells). Data are pooled from samples of 5 patients.
