# Supplementary material for: Blocking the recruitment of naive CD4+ T cells reverses immunosuppression in breast cancer
Source: Cell Res. 2017 Mar 14;27(4):461–82. doi: 10.1038/cr.2017.34 (PMC5385617; doi:10.1038/cr.2017.34)
Supplement: Supplementary information, Figure S3 — CD4+ CD45RA+ T cells in breast cancer are associated with Treg enrichment. [file cr201734x3.pdf]

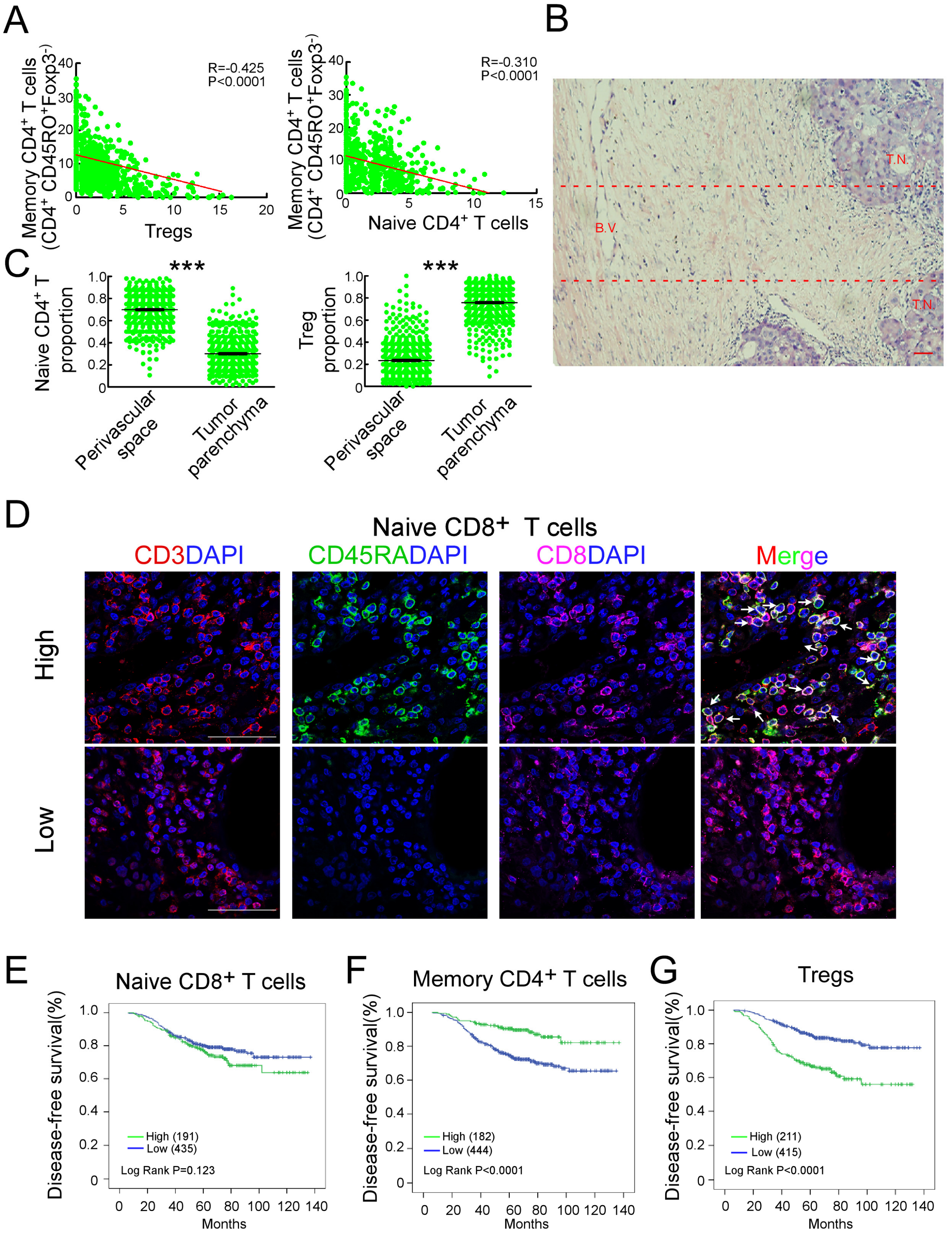

Supplementary Figure 3. CD4^{+} CD45RA^{+} T cells in breast cancer are associated with Treg enrichment.

A. Correlation of memory CD4^{+} T cells number and Treg (left) or naïve CD4^{+} T cell (right) number in breast cancer samples (n=626).

B. H&E staining of a serial section used in Figure 2C**.** The dot lines indicate the area of immunofluorescent staining showed in Figure 2C. B.V., blood vessel; T.N., tumor nest. Scale bars, 50 μm.

C. Quantitation of naïve $CD4^+$ T cell (left) or Treg (right) distribution in the space close to the blood vessels and tumor parenchyma. (n=501 for Treg quantitation, n=462 for naïve $CD4^+$ T cell quantitation; ***, $p<0.001$ by the Mann-Whitney U test)

D. Representative immunofluorescent staining of CD3 (red), CD45RA (green), CD8 (purple) and DAPI (blue) in breast cancer samples with high (upper panel) or low (lower panel) number of naïve $CD8^+$ T cells, which are indicated by arrows. Scale bars, 50 μm.

E-G. Kaplan-Meier survival curve of breast cancer patients with low and high number of tumor-infiltrating naïve $CD8^+$ T cells (**E**) , memory $CD4^+$ T cells (**F**) and Tregs (**G**).
