# Supplementary material for: Blocking the recruitment of naive CD4+ T cells reverses immunosuppression in breast cancer
Source: Cell Res. 2017 Mar 14;27(4):461–82. doi: 10.1038/cr.2017.34 (PMC5385617; doi:10.1038/cr.2017.34)
Supplement: Supplementary information, Figure S4 — CD4+ CD62L+ T cells in breast cancer are associated with poor patient prognosis. [file cr201734x4.pdf]

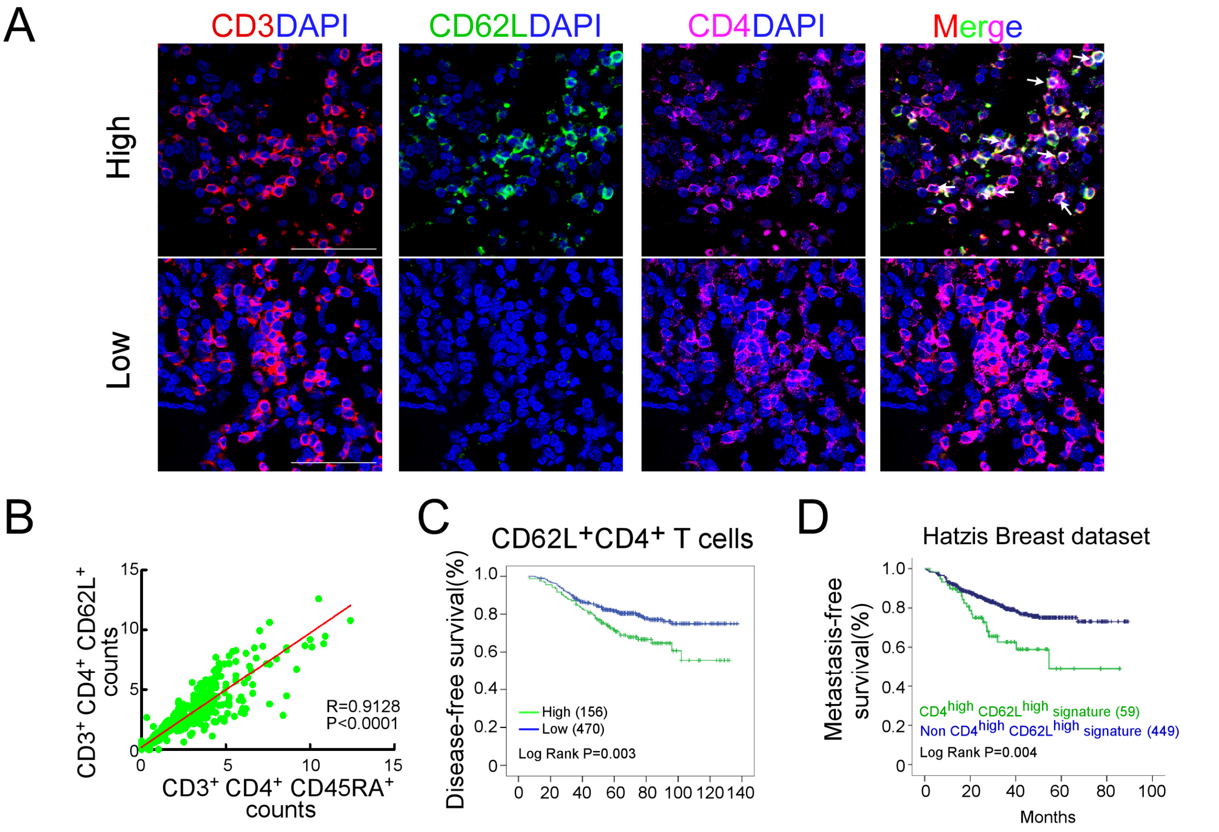

Supplementary Figure 4. $CD4^+$ $CD62L^+$ T cells in breast cancer are associated with poor patient prognosis.

A. Representative immunofluorescent staining of CD3 (red), CD62L (green), CD4 (purple) and DAPI (blue) in breast cancer samples with high (upper panel) or low (lower panel) number of naïve $CD4^+$ T cells, which are indicated by arrows. Scale bars, 50 μm.

B. Correlation of $CD3^+CD4^+CD62L^+$ cell number and $CD3^+CD4^+CD45RA^+$cell number in breast cancer samples (n=626).

C. Kaplan-Meier survival curve of breast cancer patients with low and high number of tumor-infiltrating naïve $CD4^+$ T cells denoted by $CD3^+CD4^+CD62L^+$.

D. Kaplan-Meier survival curve of breast cancer patients with $CD4^{high}$ $CD62L^{high}$ signature and non-$CD4^{high}$ $CD62L^{high}$ signature in Hatzis breast cancer online database.
