# Supplementary material for: Blocking the recruitment of naive CD4+ T cells reverses immunosuppression in breast cancer
Source: Cell Res. 2017 Mar 14;27(4):461–82. doi: 10.1038/cr.2017.34 (PMC5385617; doi:10.1038/cr.2017.34)
Supplement: Supplementary information, Figure S5 — Naïve CD4+ T cells are converted to functional Tregs by tumor-infiltrating myeloid DCs and tumor conditioned medium. [file cr201734x5.pdf]

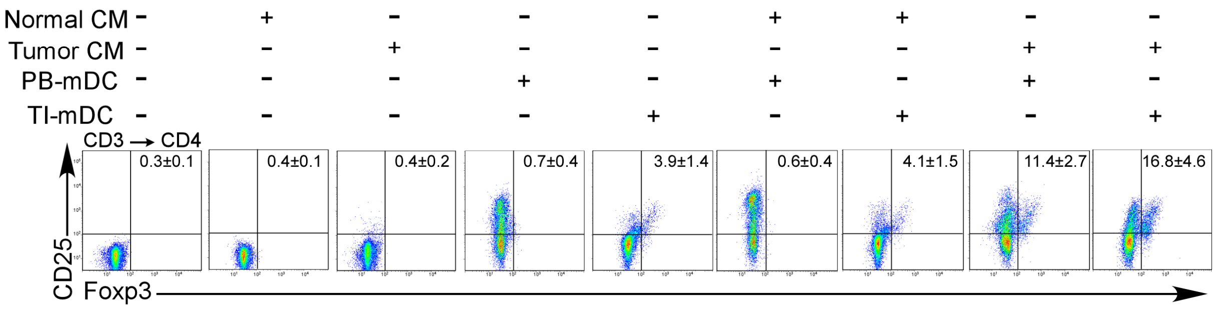

Supplementary Figure 5. Naïve CD4$^+$ T cells are converted to functional Tregs by tumor-infiltrating myeloid DCs and tumor conditioned medium.

Naïve CD4$^+$ T cells isolated from peripheral blood of patients with invasive breast carcinoma were co-cultured with or without autologous myeloid DCs isolated from tumor (TI) or peripheral blood (PB) for 9 days in the presence or absence of 30% conditioned medium (CM) from autologous tumor slices or adjacent normal tissue slices. Non-adherent cells from co-cultures were stained for CD3, CD4, CD25 and intracellular Foxp3 and analyzed by flow cytometry. Representative plots of gated CD3$^+$CD4$^+$ cells are shown (mean ± s.e.m, n=5).
