# Supplementary material for: Blocking the recruitment of naive CD4+ T cells reverses immunosuppression in breast cancer
Source: Cell Res. 2017 Mar 14;27(4):461–82. doi: 10.1038/cr.2017.34 (PMC5385617; doi:10.1038/cr.2017.34)
Supplement: Supplementary information, Figure S6 — Tregs Converted from Naïve CD4+ T Cells Suppress Tumor-associated Antigen-Specific CTLs [file cr201734x6.pdf]

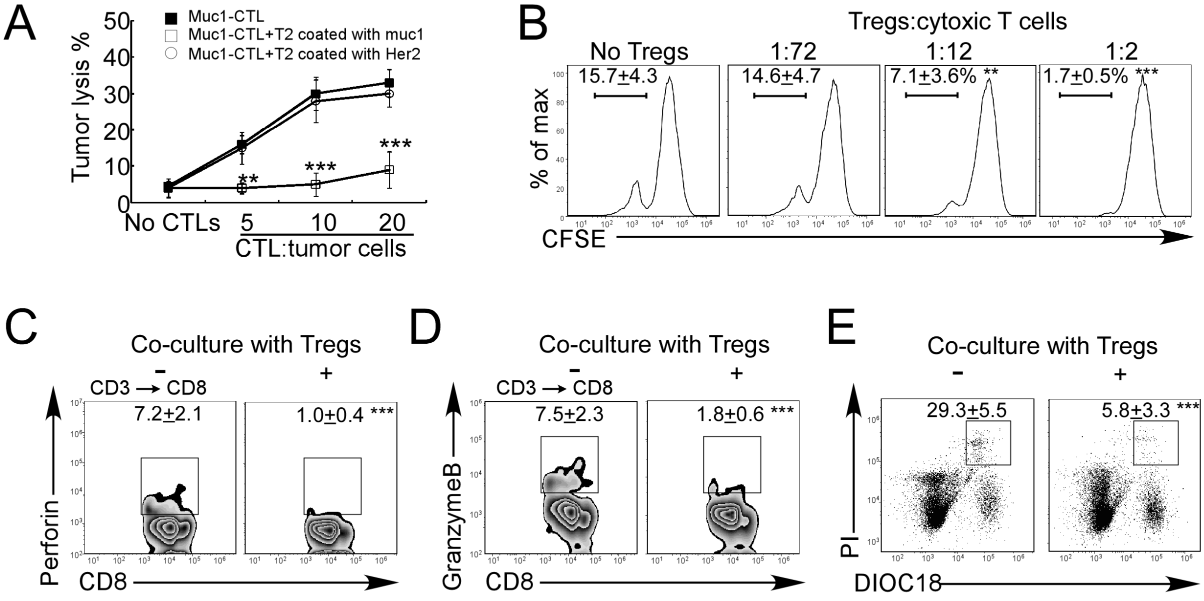

Supplementary Figure 6. Tregs Converted from Naïve CD4$^+$ T Cells Suppress Tumor-associated Antigen-Specific CTLs

Muc1-specific CTLs were generated by incubating peripheral blood CD8$^+$ T cells with Muc1-synthetic-peptide-pulsed autologous DCs derived from peripheral blood of HLA-A2$^+$ patients with tumors expressing Muc1. HLA-A2 expression in peripheral blood mononuclear cells was determined by flow cytometry. Muc1 expression in tumor was determined by IHC (data not shown).

A. DIOC18-labeled primary breast cancer cells were incubated with Muc1-specific CTLs in indicated cell ratios for 18 hr. Afterward, the death of cancer cells (PI$^+$DIOC18$^+$) was was assessed by propidium iodide (PI) uptake by flow cytometry. The Muc1 specificity of the tumor lysis was evaluated in the presence of DIOC18-unlabeled T2 cells coated with the Muc1 peptide or an irrelevant peptide at an inhibitor : target ratio of 20:1 (mean ± s.e.m, n=4; **, p<0.01; ***, p<0.001 compared to the muc1-CTL group by Student's t test). The cytotoxicity against DIOC18-labeled tumor cells was Muc1-specific as it was inhibited by DIOC18-unlabeled T2 cells coated with the Muc1 peptide, but not by those coated with an irrelevant HER-2/neu–derived peptide

B. CD4$^+$ CD25$^+$ CD127$^-$ induced Tregs were recovered from naïve CD4$^+$ T cells primed by tumor-infiltrating DCs and tumor CM by magnetic sorting. The CFSE-labeled CD8$^+$ T cells were incubated with Muc1-synthetic-peptide-pulsed autologous DCs in the presence of induced Tregs at indicated ratios and proliferation was assessed by flow cytometry. Numerical values denote the percentage of cells undergoing at least one cellular division (mean ± s.e.m, n=5, **, p<0.01,***, p<0.001 compared with CD8$^+$ T cells cultured without Tregs).

C-E. Muc1-specific CTLs were incubated with autologous primary breast cancer cells for 18 hr in the presence or absence of Tregs (CD8:Treg 2:1) and stained for CD3, CD8, intracellular perforin (**C**) or granzyme B (**D**) and gated CD3$^+$CD8$^+$ cells were analyzed by flow cytometry. Numbers indicate mean ± s.e.m % of gated cells that stained for perforin or granzyme B (mean ± s.e.m, n=4, ***, p<0.001 compared with CTLs cultured without Tregs).

E. Muc1-specific CTLs were incubated with autologous DIOC18-labeled primary breast cancer cells for 18 hr in the presence or absence of Tregs (CD8:Treg 2:1) and the death of tumor cells was assessed by propidium iodide (PI) uptake by flow cytometry. The numbers shown indicate the mean ± s.e.m percentage of PI^+ tumor cells (mean ± s.e.m, n=4, ***, $p<0.001$ compared with CTLs cultured without Tregs)
