# Supplementary material for: Blocking the recruitment of naive CD4+ T cells reverses immunosuppression in breast cancer
Source: Cell Res. 2017 Mar 14;27(4):461–82. doi: 10.1038/cr.2017.34 (PMC5385617; doi:10.1038/cr.2017.34)
Supplement: Supplementary information, Figure S7 — Naïve CD4+ T cells are recruited to breast tumors by TAM-secreted CCL18. [file cr201734x7.pdf]

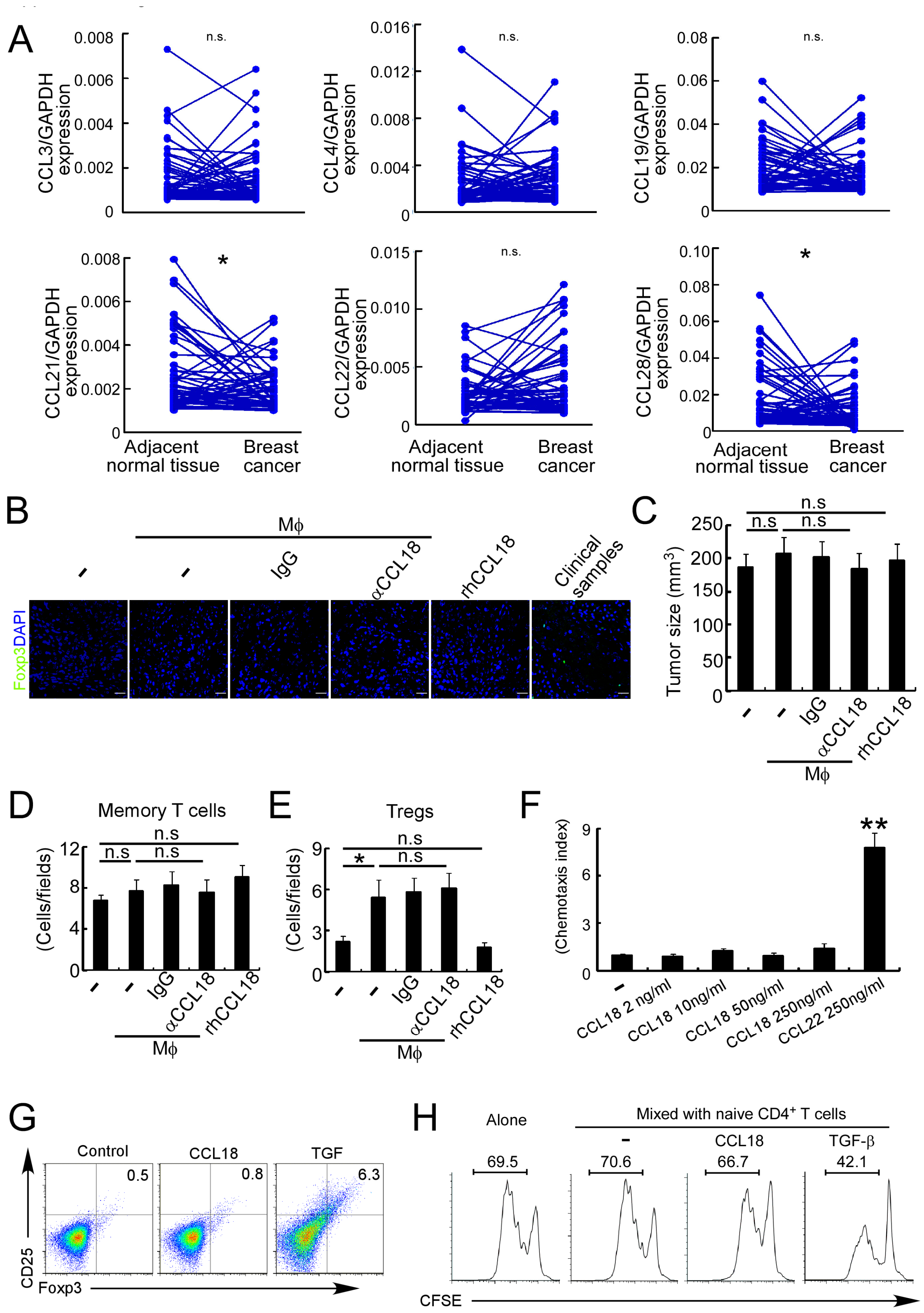

Supplementary Figure 7. Naïve CD4 $^{+}$ T cells are recruited to breast tumors by TAM-secreted CCL18.

A. CCL3,CCL4,CCL19,CCL21,CCL22 and CCL28 expression in breast cancer tissues and paired adjacent normal breast tissues detected by qPR-PCR relative to *GAPDH* (n=52, *,

p<0.05; n.s. , non significant by Student's t test).

B. & C. Treg infiltration in xenografts determined by immunostaining for Foxp3 (**B**) and tumor sizes (**C**) of mice treated as in Figure. 4E. (mean ± s.e.m. n=8 for each group. n.s. , non significant by Student's t test. Scale bars, 50μm).A section of clinical sample with Treg infiltration was used as the positive control for Foxp3 staining.

D. & E. CFSE-labeled memory $CD4^+$ T cells (**D**) or Tregs(**E**) from healthy donors were intravenously injected via tail vein, with or without control IgG or CCL18-neutralizing antibody, into NOD/scid mice bearing subcutaneous MDA-MB-231 breast cancers that were implanted 14 d earlier either alone or with autologous human macrophages. In some mice, the xenografts were injected with rhCCL18. The xenografts were harvested after 48 hr and the migrated T cell number in xenografts were quantitated (mean ± s.e.m. n=8 for each group. *, p<0.05; n.s. , non significant by Student's t test).

F. Blunted chemotaxis of Tregs isolated from peripheral blood of healthy donors to CCL18 or CCL22 in a Transwell assay Shown are mean ± s.e.m. chemotaxis indices for 3 independent experiments. (**, p<0.01 by Student's t-test).

G & H. Naïve $CD4^+$ T cells isolated from the peripheral blood of healthy donors were treated with anti-CD3, anti-CD28 and CCL18 or TGF-β for 9 days

G. The cells were stained for CD3, CD4, CD25 and intracellular Foxp3 and analyzed by flow cytometry. Representative flow plots were presented (n=4). Cells were gated on the $CD3^+CD4^+$ population.

H. The cells were incubated with CFSE-stained autologous $CD8^+$ cytotoxic T cells that were stimulated with 231 cell lysate-pulsed myeloid DCs in a suppression assay. The flow plots are representative of independent experiments of 4 donors. Numerical values denote the percentage of cells undergoing at least one cellular division
