# Supplementary material for: Blocking the recruitment of naive CD4+ T cells reverses immunosuppression in breast cancer
Source: Cell Res. 2017 Mar 14;27(4):461–82. doi: 10.1038/cr.2017.34 (PMC5385617; doi:10.1038/cr.2017.34)
Supplement: Supplementary information, Figure S8 — PITPNM3 is expressed in naïve CD4+ T cells. [file cr201734x8.pdf]

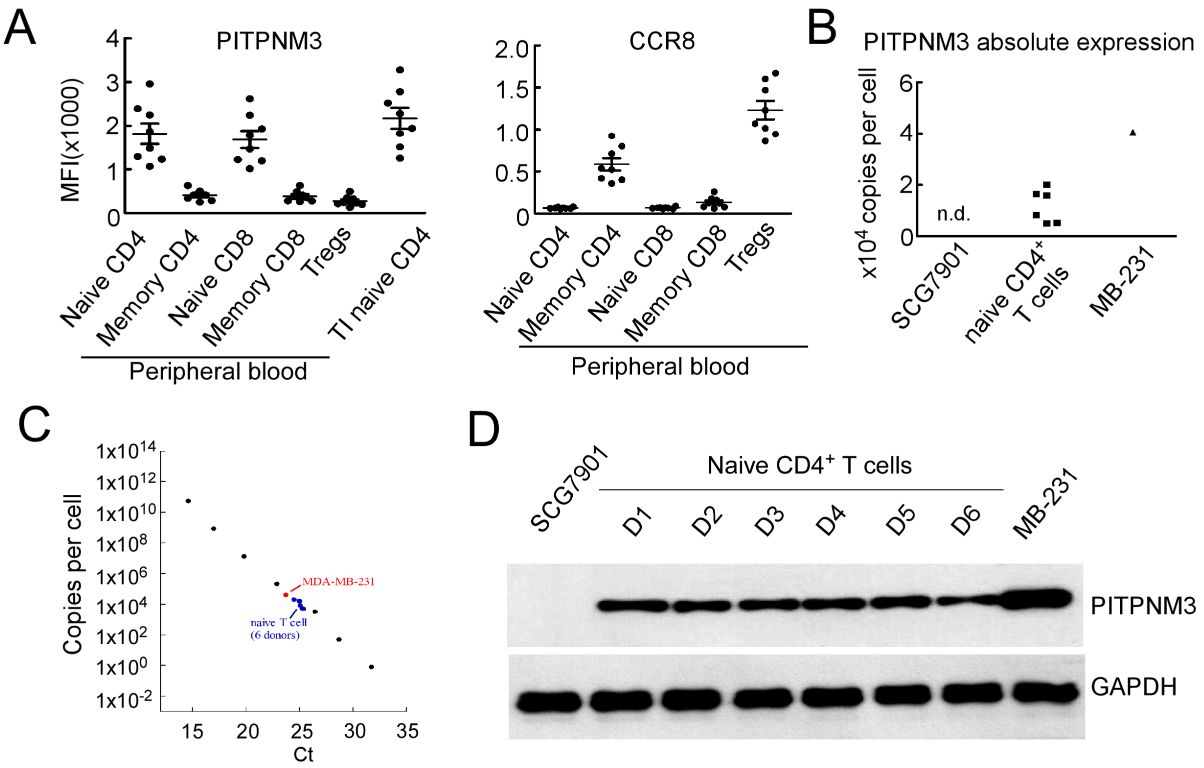

Supplementary Figure 8. PITPNM3 is expressed in naïve CD4$^+$ T cells.

A. Quantitation of MFIs in Figure 5A (n=8)

B. Naïve CD4$^+$ T cells were purified from peripheral blood of 6 healthy donors and detected for PITPNM3 mRNA expression by absolute qRT-PCR. The dots represent the average of triplicate experiments from the same donor. n.d., not detectable.

C. Estimated copy numbers of PITPNM3 mRNA per naive CD4$^+$ T cells or MDA-MB-231 cells. Equivalent molecules per cell were calculated based on the assumption that total RNA per cell is 20 pg.

D. Naïve CD4$^+$ T cells were purified from peripheral blood of 6 healthy donors and detected for PITPNM3 protein level by western blots**.** SCG7901 cells and MDA-MB-231 cells were used as the negative control and positive control, respectively.
