# Supplementary material for: Blocking the recruitment of naive CD4+ T cells reverses immunosuppression in breast cancer
Source: Cell Res. 2017 Mar 14;27(4):461–82. doi: 10.1038/cr.2017.34 (PMC5385617; doi:10.1038/cr.2017.34)
Supplement: Supplementary information, Figure S9 — CD4 aptamir-PITPNM3 siRNA reverses immunosuppression and inhibits tumor progression in humanized mice. [file cr201734x9.pdf]

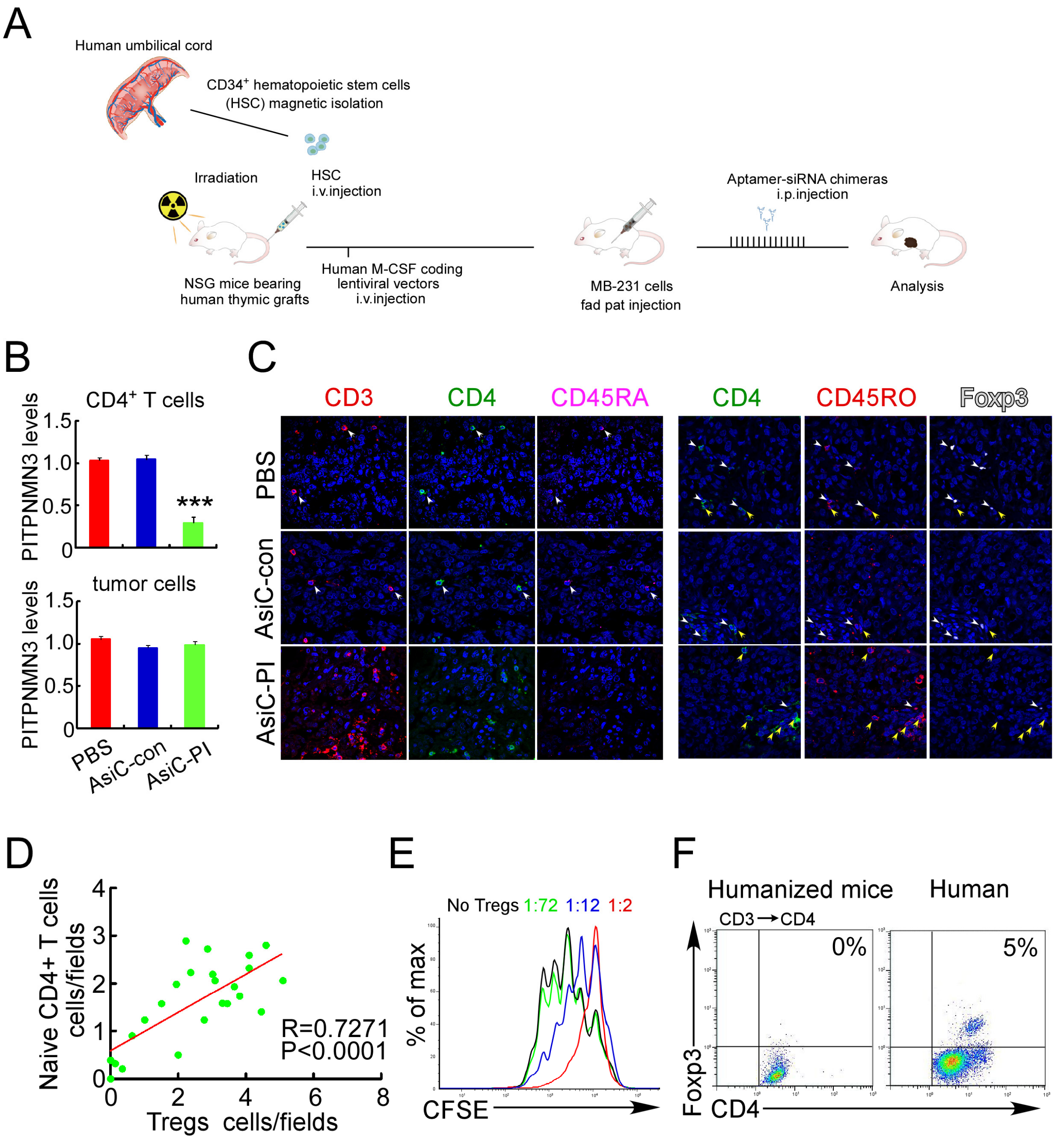

Supplementary Figure 9. CD4 aptamir-PITPNM3 siRNA reverses immunosuppression and inhibits tumor progression in humanized mice.

Humanized mice bearing palpable MDA-MB-231 orthotopic xenografts were intraperitoneally injected daily for 14 d with PBS, 1 nmol CD4-aptamer–control siRNA (AsiC-con) or CD4-aptamer–siRNA targeting *PITPNM3* to assess the role of PITPNM3 on TI Tregs and other T cells and tumor control.

A. Experimental schematic

B. The human CD4$^+$ T cells and MDA-MB-231 cells were isolated from peripheral blood and xenografts, respectively. Their RNA levels of PITPNM3 were detected by qRT-PCR relative to *GAPDH* (Mean ± s.e.m. n=8 for each group; ***, p<0.001 by Student's t test).

C. The pictures of separate channels of the triple immunofluorescent staining images in

Figure. 6D. Naive $CD4^+$ T cells ($CD3^+CD4^+$ $CD45RA^+$ left panel) and Tregs ($CD4^+$ $Foxp3^+$ right panel) were indicated by white arrowheads. Memory $CD4^+$ T cells ($CD4^+CD45RO^+$ $Foxp3^-$ right panel) were indicated by yellow arrows.

D. Correlation of naive $CD4^+$ T cell number and Treg number in xenografts (n=24)

E. CFSE-labeled $CD8^+$ T cells isolated from peripheral blood were treated with anti-CD3 and anit-CD28 in the presence of $CD4^+CD25^+CD127^-$ Tregs isolated from tumors by magnetic sorting at indicated ratios and proliferation was assessed by flow cytometry. The flow plot was a representative of independent experiments of 6 humanized mice.

F. $CD4^+$ T cells were isolated from peripheral blood of humanized mice or healthy human donors and stained for human CD3, CD4 and intracellular for Foxp3 and analyzed by flow cytometry.$CD3^+$ $CD4^+$-gated populations were further evaluated for Foxp3 levels. The flow plots were representatives of independent experiments of 6 humanized mice.
