# Supplementary material for: Blocking the recruitment of naive CD4+ T cells reverses immunosuppression in breast cancer
Source: Cell Res. 2017 Mar 14;27(4):461–82. doi: 10.1038/cr.2017.34 (PMC5385617; doi:10.1038/cr.2017.34)
Supplement: Supplementary information, Table S1 — Correlation between tumor infiltrating naïve CD4+ T cells and clinicopathological parameters [file cr201734x10.pdf]

Supplementary Table S1. Correlation between tumor infiltrating naïve CD4$^+$ T cells and clinicopathological parameters

	Low naïve CD4$^+$ T cell count		High naïve CD4$^+$ T cell count		
**Factor**	No.	%	No.	%	*P* value
All patients	513	81.9	113	18.1	
Age ,years					0.522
≤45	195	80.6	47	19.4	
>45	318	82.8	66	17.2	
menopause					0.917
Premenopause	280	82.1	61	17.9	
Promenopause	233	81.6	52	18.4	
Tumor size, cm					0.063
≤2	185	86.0	30	14.0	
>2	328	79.8	83	20.2	
Node status					0.028*
Negative	226	85.9	37	14.1	
Positive	287	79.1	76	20.9	
Tumor grade					0.065
1 or 2	362	82.5	77	17.5	
3	151	80.7	36	19.3	
Lymphovascular invasion					0.046*
Negative	446	83.7	87	16.3	
Positive	53	73.6	19	26.4	
Missing	14	66.7	7	33.3	
Stage					0.081
Ⅰ–Ⅱ	379	83.7	74	16.3	
Ⅲ	134	77.5	39	22.5	
ER status					0.079

Negative	167	78.0	47	22.0	
Positive	346	83.9	66	16.1	
PR status					0.243
Negative	199	79.6	51	20.4	
Positive	314	83.5	62	16.5	
Her2 status					1.000
Negative	343	81.9	76	18.1	
Positive	170	82.1	37	17.6	
Tumor subtype					0.018*
HR^+ $Her2^-$	259	85.2	45	14.8	
HR^+Her2^+	93	83.0	19	17.0	
HR^-Her2^+	76	80.9	18	19.1	
HR^-Her2^-	85	73.3	31	36.7	
Distant metastasis					0.007*
Negative	405	84.4	75	15.6	
Positive	108	73.9	38	26.1	

Abbreviations: ER, estrogen receptor; PR, progesterone receptor; HER2, human epidermal growth factor receptor; HR, hormone receptor.

Distant metastasis identified during postoperative follow-up.
