# Supplementary material for: Blocking the recruitment of naive CD4+ T cells reverses immunosuppression in breast cancer
Source: Cell Res. 2017 Mar 14;27(4):461–82. doi: 10.1038/cr.2017.34 (PMC5385617; doi:10.1038/cr.2017.34)
Supplement: Supplementary information, Table S2 — Cox regression analysis of DFS in entire cohort (n=626) [file cr201734x11.pdf]

**Supplementary Table S2.**Cox regression analysis of DFS in entire cohort (n=626)

| | DFS in entire cohort | | | |
|---|---|---|---|---|
| | Univariate | Multivariate | | |
| | P | HR | 95% CI | P |
| Age (＞45) | 0.813 | | | |
| Premenopause | 0.177 | | | |
| Tumor size (＞2cm) | <0.0001 | 1.834 | 1.203-2.795 | 0.005* |
| Lymph node (positive) | <0.0001 | 1.960 | 1.319-2.912 | 0.001* |
| Grade (III ) | 0.024 | 1.327 | 0.946-1.862 | 0.102 |
| LVI (positive) | 0.204 | | | |
| ER (positive) | 0.004 | 0.951 | 0.626-1.553 | 0.951 |
| PR (positive) | 0.001 | 0.683 | 0.435-1.071 | 0.097 |
| Her2 (positive) | 0.001 | 1.511 | 1.081-2.110 | 0.016* |
| Naïve $CD4^{+}$ T cells (high) | 0.006 | 1.469 | 1.009-2.138 | 0.041* |

Abbreviations: ER, estrogen receptor; PR, progesterone receptor; HER2, human epidermal growth factor receptor. 1
