# Supplementary material for: Blocking the recruitment of naive CD4+ T cells reverses immunosuppression in breast cancer
Source: Cell Res. 2017 Mar 14;27(4):461–82. doi: 10.1038/cr.2017.34 (PMC5385617; doi:10.1038/cr.2017.34)
Supplement: Supplementary information, Table S3 — Correlation of CD4+ naïve T cell associated gene expression and clinical features in breast cancer in the Oncomine online database [file cr201734x12.pdf]

Supplementary Table S3. Correlation of $CD4^+$ naïve T cell associated gene expression and clinical features in breast cancer in the Oncomine online database

**Factor**	**No.**	**%**	**No.**	**%**	**P value**	**Database(case number)**
	**Non-$CD4^{high}CD62L^{high}$ (1015)**		**$CD4^{high}CD62L^{high}$ (924)**			**Curtis Breast(1939)**
Node status					0.037*	
0-3	869	53.4	759	46.6		
⩾4	146	46.9	165	53.1		
Tumor subtype					<0.0001*	
Non-TNBC	929	55.6	742	44.4		
TNBC	86	32.1	182	67.9		
	**Non-$CD4^{high}CD62L^{high}$ (449)**		**$CD4^{high}CD62L^{high}$ (59)**			**Hatzis Breast(508)**
Node status					0.010*	
0-3	362	90.3	39	9.7		
⩾4	87	81.3	20	18.7		
Tumor subtype					0.105	
Non-TNBC	292	90.1	32	9.9		
TNBC	157	85.3	27	14.7		
Metastasis					0.007*	
No	359	90.4	38	9.6		
Yes	90	81.1	21	18.9		

Notes: CD62L is designated as SELL in Oncomine. The information of lymphovascular invasion is absent in both datasets and the information of distant metastasis is absent in Hazis dataset.
