# Supplementary material for: Blocking the recruitment of naive CD4+ T cells reverses immunosuppression in breast cancer
Source: Cell Res. 2017 Mar 14;27(4):461–82. doi: 10.1038/cr.2017.34 (PMC5385617; doi:10.1038/cr.2017.34)
Supplement: Supplementary information, Table S4 — T cell chemotactic cytokine expression in breast cancer in the online databases [file cr201734x13.pdf]

Supplementary Table S4. T cell chemotactic cytokine expression in breast cancer in the online databases

Datasets	Tissues(case number)	Gene	Fold change	p-value
GSE53752	Invasive Ductal Carcinoma (53) vs Normal Human Breast Tissue (25)	CCL3	1.08	0.15
		CCL4	1.07	0.08
		CCL18	1.33	<0.001*
		CCL19	0.79	0.06
		CCL21	0.66	0.018*
		CCL22	0.56	0.007*
		CCL28	0.55	<0.001*
GSE38959	Invasive Ductal Carcinoma (30) VS Normal Human Breast Tissue (13)	CCL3	1.64	0.28
		CCL4	3.15	0.07
		CCL18	35.78	0.03*
		CCL19	2.75	0.20
		CCL21	1.29	0.30
		CCL22	1.36	0.07
		CCL28	0.105	<0.001*
GSE15852	Invasive Ductal Carcinoma (43) vs Normal Human Breast Tissue (43)	CCL3	0.94	0.02*
		CCL4	0.94	0.04*
		CCL18	1.28	0.66
		CCL19	1.25	0.11
		CCL21	1.17	0.33
		CCL22	1.21	0.26
GSE33487	Invasive Ductal Carcinoma (6) vs Normal Human Breast Tissue (2)	CCL3	5.04	0.18
		CCL4	4.38	0.33
		CCL18	8.31	0.17
		CCL19	2.42	0.51
		CCL21	0.20	0.03*
		CCL22	1.73	0.29
		CCL28	0.35	0.12
GSE10797	Invasive Ductal Carcinoma (28) vs Normal Human Breast Tissue (5)	CCL3	0.98	0.69
		CCL4	1.08	0.17
		CCL18	1.19	0.08
		CCL19	1.23	0.19
		CCL21	0.99	0.92
		CCL22	1.07	0.34

CCL28 expression data are not available in GSE15852 and GSE10797.
