# Supplementary material for: Blocking the recruitment of naive CD4+ T cells reverses immunosuppression in breast cancer
Source: Cell Res. 2017 Mar 14;27(4):461–82. doi: 10.1038/cr.2017.34 (PMC5385617; doi:10.1038/cr.2017.34)
Supplement: Supplementary information, Table S5 — Cardiac and liver functions in humanized mice injected with aptamer-siRNA chimeras [file cr201734x14.pdf]

Supplementary Table S5. Cardiac and liver functions in humanized mice injected with aptamer-siRNA chimeras

	CK (U/L)	LDH (U/L)	CK-MB (U/L)	ALT (U/L)	Total bilirubin (μmol/L)	Direct bilirubin (μmol/L)
PBS	653.4±105.2	512.9±135.9	643.2±112.6	20.1±3.5	0.95±0.21	0.49±0.12
AsiC-con	637.8±135.7	528.7±98.6	602.5±104.8	18.4±5.2	0.97±0.18	0.47±0.13
AsiC-PI	649.5±128.4	543.6±126.7	627.4±146.7	21.2±6.3	0.94±0.23	0.43±0.16

Abbreviations: CK, creatine kinase; LDH, lactate dehydrogenase; CK-MB, creatine kinase–muscle and brain; ALT, alanine transaminase.
