# Supplementary material for: Blocking the recruitment of naive CD4+ T cells reverses immunosuppression in breast cancer
Source: Cell Res. 2017 Mar 14;27(4):461–82. doi: 10.1038/cr.2017.34 (PMC5385617; doi:10.1038/cr.2017.34)
Supplement: Supplementary information, Table S6 — The PCR primer sequences [file cr201734x15.pdf]

Supplementary Table S6. The PCR primer sequences

GAPDH	5′-ATCACCATCTTCCAGGAGCGA-3′(forward)
	5′-CCTTCTCCATGGTGGTGAAGAC-3′(reverse)
PITPNM3	5′-GTTCCAAGCAGCAATCAGGT-3' (forward)
	5' -TCACAATATCTCACCGTACACCA -3' (reverse)
CCL3	5′-TTCTTGGCTCTGCTGACACTC-3' (forward)
	5′-CACTGGCTGCTCGTCTCAA -3' (reverse)
CCL4	5′-CGTGTATGACCTGGAACTGAAC-3' (forward)
	5′-AGGAACTGCGGAGAGGAGTC -3' (reverse)
CCL18	5′-CTCTGCTGCCTCGTCTATACCT-3' (forward)
	5′-CTTGGTTAGGAGGATGACACCT -3' (reverse)
CCL19	5′-CCAGCCCCAACTCTGAGTG-3' (forward)
	5′-ATCCTTGATGAGAAGGTAGTGGA -3' (reverse)
CCL21	5′-AGCCTCCTTATCCTGGTTCTG-3' (forward)
	5′-ACAACCTTGGCGGGAATCTTC -3' (reverse)
CCL22	5′-ATCGCCTACAGACTGCACTC-3' (forward)
	5′-GACGGTAACGGACGTAATCAC -3' (reverse)
CCL28	5′-TGCACGGAGGTTTCACATCAT-3' (forward)
	5′-ACAGATTCTTCTGCGCTTGAC -3' (reverse)
TGF-β1	5′-AAGGACCTCGGCTGGAAGTGC-3' (forward)
	5′-CCGGGTTATGCTGGTTGTA -3' (reverse)
IL-10	5′-AACAAGAGCAAGGCCGTGG-3' (forward)
	5′-GAAGATGTCAAACTCACTCATGGC -3' (reverse)
CTLA4	5′-CTACCTGGGCATAGGC AACG-3' (forward)
	5′-CCCCGAACTAACTGCTGCAA -3' (reverse)
GITR	5′-TGCAAACCTTGGACAGACTGC-3' (forward)
	5′-ACAGCGTTGTGGGTCTTGTTC -3' (reverse)
HPRT	5′-TTCCTTGGTCAGGCAGTATAATCC -3′(forward)
	5′-AGTCTGGCTTATATCCAACACTTCG -3′(reverse)
18S rRNA	5′- CGGCTACCACATCCAAGGAA -3′(forward)
	5′- GCTGGAATTACCGCGGCT -3′(reverse)
PGMA1-TRBV12-4	5′-CCATCTCATCCCTGCGTGTCTCCGACTCAGCTAAGGTAACGAT***CTGAAGATCCAGCCCTCAG***-3' (forward)
PGMA2-TRBV12-4	5′-CCATCTCATCCCTGCGTGTCTCCGACTCAGTAAGGAGAACGAT***CTGAAGATCCAGCCCTCAG***-3' (forward)
PGMA3-TRBV12-4	5′-CCATCTCATCCCTGCGTGTCTCCGACTCAGAAGAGGATTCGAT***CTGAAGATCCAGCCCTCAG***-3' (forward)
PGMA4-TRBV12-4	5′-CCATCTCATCCCTGCGTGTCTCCGACTCAGTACCAAGATCGAT***CTGAAGATCCAGCCCTCAG***-3' (forward)
PGMA5-TRBV12-4	5′-CCATCTCATCCCTGCGTGTCTCCGACTCAGCAGAAGGAACGAT***CTGAAGATCCAGCCCTCAG***-3' (forward)
PGMA6-TRBV12-4	5′-CCATCTCATCCCTGCGTGTCTCCGACTCAGCTGCA

	AGTTCGAT***CTGAAGATCCAGCCCTCAG***-3' (forward)
PGMA7-TRBV12-4	5′-CCATCTCATCCCTGCGTGTCTCCGACTCAGTTCGTGATTCGAT***CTGAAGATCCAGCCCTCAG***-3' (forward)
PGMA8-TRBV12-4	5′-CCATCTCATCCCTGCGTGTCTCCGACTCAGTTCCGATAACGAT***CTGAAGATCCAGCCCTCAG***-3' (forward)
PGMA9-TRBV12-4	5′-CCATCTCATCCCTGCGTGTCTCCGACTCAGTGAGCGGAACGAT***CTGAAGATCCAGCCCTCAG***-3' (forward)
PGMA10-TRBV12-4	5′-CCATCTCATCCCTGCGTGTCTCCGACTCAGCTGACCGAACGAT***CTGAAGATCCAGCCCTCAG***-3' (forward)
PGMA11-TRBV12-4	5′-CCATCTCATCCCTGCGTGTCTCCGACTCAGTCCTCGAATCGAT***CTGAAGATCCAGCCCTCAG***-3' (forward)
PGMA12-TRBV12-4	5′-CCATCTCATCCCTGCGTGTCTCCGACTCAGTAGGTGGTTCGAT***CTGAAGATCCAGCCCTCAG***-3' (forward)
PGMP-TRBJ1-2	5′-TCCGCTTTCCTCTCTATGGGCAGTCGGTGAT***GTTAACCTGGTCCCCGAAC***-3' (reverse)
Multiplexing PCR Primer (P5)	5′-AATGATACGGCGACCACCGAGATCTACACTCTTTCCCTACACGACGCTCTTCCGATCT -3′
Multiplexing PCR Primer (P7)-1	5′-CAAGCAGAAGACGGCATACGAGATCATTGCTTGACTGGAGTTCAGACGTGTGCTCTTCCGATCT -3′
Multiplexing PCR Primer (P7)-2	5′-CAAGCAGAAGACGGCATACGAGATTTCGGATTGACTGGAGTTCAGACGTGTGCTCTTCCGATCT -3′
Multiplexing PCR Primer (P7)-3	5′-CAAGCAGAAGACGGCATACGAGATTCATCATTGACTGGAGTTCAGACGTGTGCTCTTCCGATCT -3′
Multiplexing PCR Primer (P7)-4	5′-CAAGCAGAAGACGGCATACGAGATGCTCCTGTGACTGGAGTTCAGACGTGTGCTCTTCCGATCT -3′
Multiplexing PCR Primer (P7)-5	5′-CAAGCAGAAGACGGCATACGAGATAGCTCGGTGACTGGAGTTCAGACGTGTGCTCTTCCGATCT -3′
Multiplexing PCR Primer (P7)-6	5′-CAAGCAGAAGACGGCATACGAGATCAACAGGTGACTGGAGTTCAGACGTGTGCTCTTCCGATCT -3′
Multiplexing PCR Primer (P7)-7	5′-CAAGCAGAAGACGGCATACGAGATTTCAAGGTGACTGGAGTTCAGACGTGTGCTCTTCCGATCT -3′
Multiplexing PCR Primer (P7)-8	5′-CAAGCAGAAGACGGCATACGAGATCCTAACGTGACTGGAGTTCAGACGTGTGCTCTTCCGATCT -3′
Multiplexing PCR Primer (P7)-9	5′-CAAGCAGAAGACGGCATACGAGATCACGTAGTGACTGGAGTTCAGACGTGTGCTCTTCCGATCT -3′
P5 Multiplexing Adapters	5'-ACACTCTTTCCCTACACGACGCTCTTCCGATCT-3′
P7 Multiplexing Adapters	5′-P-GATCGGAAGAGCACACGTCT-3′

For each sample, we amplified the gene using the forward primer: 5′-CCATCTCATCCCTGCGTGTCTCCGACTCAGNNNNNNNNNCGAT***CTGAAGATCCAGCCCTCAG***-3' (forward), the bold sequences are TRBV12-4 primers, the underlined sequences were designed to tag PCR products of each sample as barcodes, the sequence before underlined sequences is adaptor primer for sequencing, and "CGAT" designed as a linker between the barcodes and TRBV12-4 primer. The reverse primer is:

5′-TCCGCTTTCCTCTCTATGGGCAGTCGGTGAT***GTTAACCTGGTCCCCGAAC***-3'(reverse), the bold sequences is TRBJ1-2 primer, before bold sequences is adaptor primer for sequence. The library of full length TCR sequence was constructed by using Multiplexing PCR Primer P5 and P7.
